# Modification of sugarcane bagasse with iron(III) oxide-hydroxide to improve its adsorption property for removing lead(II) ions

**DOI:** 10.1038/s41598-023-28654-5

**Published:** 2023-01-26

**Authors:** Pornsawai Praipipat, Pimploy Ngamsurach, Amornrat Sanghuayprai

**Affiliations:** 1 Department of Environmental Science, Khon Kaen University, Khon Kaen, 40002 Thailand; 2 Environmental Applications of Recycled and Natural Materials (EARN) Laboratory, Khon Kaen University, Khon Kaen, 40002 Thailand

**Keywords:** Chemical engineering, Engineering, Materials science

## Abstract

Lead contamination in wastewater results in toxicity of aquatic life and water quality, it is recommended to remove lead before discharging. Four sugarcane bagasse adsorbent materials of sugarcane bagasse powder (SB), sugarcane bagasse powder doped iron(III) oxide-hydroxide (SBF), sugarcane bagasse powder beads (SBB), and sugarcane bagasse powder doped iron(III) oxide-hydroxide beads (SBFB) were synthesized and characterized with various techniques. Their lead removal efficiencies were investigated by batch experiments on the effects of dose (0.1–0.6 g), contact time (1–6 h), pH (1, 3, 5, 7, 9, 11), and concentration (5–30 mg/L), adsorption isotherms, kinetics, and desorption experiments. All materials were amorphous phases presenting specific peaks of cellulose. SBB and SBFB detected sodium alginate peaks, and iron(III) oxide-hydroxide peaks were detected in SBF and SBFB. SB and SBF were scales or overlapping plate surfaces whereas SBB and SBFB had spherical shapes with coarse surfaces. The main functional groups of O–H, C=O, C–H, C–O, and C=C were observed in all materials, whereas Fe–O and –COOH were only found in materials with adding iron(III) oxide-hydroxide or bead material. The point of zero charges (pH_pzc_) of all materials was higher than 4. The optimum conditions of SB, SBF, SBB, and SBFB with the highest lead removal efficiency at a lead concentration of 10 mg/L and pH 5 were 0.6 g and 6 h (96.08%), 0.2 g and 3 h (100%), 0.2 g and 2 h (98.22%), and 0. 1 g and 2 h (100%), respectively. Since SBFB spent less adsorbent dose and contact time than other materials with a lead removal efficiency of 100%, it was a more potential adsorbent than other materials. Thus, adding iron(III) oxide-hydroxide and changing material form helped to improve material efficiencies for lead adsorption. The maximum adsorption capacities of SB, SBF, SBB, and SBFB were 6.161, 27.027, 23.697, and 57.471 mg/L, respectively by fitting the Langmuir model. Langmuir isotherm was best fitted for SB and SBB, whereas the Freundlich model was best fitted for SBF and SBFB. The pseudo-second-order kinetic model was best fitted for all materials. Moreover, all adsorbents could be reused for more than 5 cycles with the lead removal efficiency of more than 73%. Therefore, SBFB was potential material to further apply for lead removal in industrial applications.

## Introduction

The contaminated heavy metals in receiving water are a concern because of their bioaccumulation, persistence, and toxicity to aquatic life and the environment^1^. Lead (Pb) is known as the top three most toxic heavy metals followed by mercury (Hg) and cadmium (Cd), and its toxicity creates many human health effects to dysfunction of many human systems such as nerve, blood, tissue, reproductive, digestive, respiratory and many diseases such as anemia, paralysis, barren, and cancer^2^. Increasing lead accumulations in receiving water may be from the discharging of many industries such as batteries, steels, dyes, pigments, and plastics from their manufacturing processes. Therefore, the wastewater with lead contamination is required to remove lead to be below the USEPA water quality standards at 0.05 and 0.2 mg/L for drinking water and industrial wastewater for a safe environment.

Conventional methods of coagulation–flocculation, chemical precipitation, reverse osmosis, membrane filtration, ion exchange, and electrochemical are used for eliminating heavy metals in wastewater; however, these methods have limitations of incomplete heavy metal removals, complicated operations with expensive cost, toxic sludges with requiring disposal^3^. As a result, many studies attempted to investigate an alternative method with an effective and environmentally friendly approach to solve this problem. The adsorption method is an interesting choice for heavy metal removals because it is an effective and eco-friendly method, easy operation, low operation cost, and low creation of sludge after treatment^4^. In addition, various adsorbents such as activated carbon, chitosan, zeolite, silica, resin, nanocomposite, tomato peels, shrimp shells, coconut shells or husk, oil palm shells, banana peels, corn stalk, rice husk, sawdust, bagasse, bagasse fly ash are available choices for eliminating specific target metal ions in wastewater^5–8^. However, this study will focus on agricultural wastes because of their benefits as low-cost adsorbents used for improving water quality along with reducing waste volumes in terms of waste management. Table 1 demonstrated the elimination of heavy metals from wastewater from various agricultural wastes of sugarcane bagasse, rice straw, coconut, corn, peanut, and sawdust. For sugarcane bagasse, it has been used for removing various heavy metals of lead (Pb), nickel (Ni), chromium (Cr), and copper (Cu) by using unmodified or modified sugarcane bagasse by a hydrothermal method for producing biochar or the addition of zinc chloride (ZnCl_2_) into activated carbon^9–12^. In addition, unmodified agave bagasse has been used for removing Pb(II), cadmium (Cd), and zinc (Zn)^13^. Many previous studies are also used unmodified sugarcane bagasse for removing various heavy metal ions of Pb(II), Ni(II), Cd(II), and Cr(II) in an aqueous solution^9,14^. Moreover, rice straw modified to biochar with nitro reduction is used for Cd(II) removal^15^. For coconut, coconut coir with sodium hydroxide (NaOH) pretreatment is also used for removing Cu(II) while coconut shell was synthesized to activated carbon with lanthanum for Cr(II) removal^16,17^. The modifications of corncob with xanthate and silica nanocomposite are used for eliminating Pb(II)^18,19^. Furthermore, the chemical modification of corn husk leaves by bismuthiol I is used for mercury (Hg) removal^20^. The peanut hull modified with iron chloride and hexamethylendiamine could be used for Cr(II) removal^21^. For sawdust, various heavy metals of Pb(II), Cu(II), and Ni(II) are removed by sawdust modifications by poly (*N*-acryloyl-l-histidine) and chitosan nanocomposite beads^22,23^. For paddy husk, it is used for producing biochar for Pb(II), Cu(II), and Zn(II) removals^24^. Among those adsorbents, since bagasse has been popularly used for removing heavy metals in wastewater because of the good chemical properties of cellulose, hemicellulose, lignin, carboxyl, or hydroxyl groups, it is a good choice for lead removal in wastewater. In addition, using sugarcane bagasse can reduce a huge of sugar factory waste along with using these wastes for improving water quality which is a valuable use of resources in an environmental aspect. Although sugarcane bagasse has good chemical properties for removing lead in an aqueous solution, the material improvement method needs to investigate for increasing lead removal efficiency in case of high-strength lead concentration in industrial applications.

**Table 1:** The elimination of heavy metals from wastewater from various agriculture wastes.

Adsorbents	Modifications	Metals	Removal efficiency	*q*_m_ (mg/g)	Refs
Sugarcane bagasse	Unmodified	Pb	89.31%	1.61	^9^
	Unmodified	Ni	96.33%	123.46	^9^
	Biochar	Pb	75.38%	12.74	^10^
	ZnCl_2_	Cr	93.61%	277.78	^11^
	ZnCl_2_-activated carbon	Pb	99.90%	19.30	^12^
	ZnCl_2_-activated carbon	Ni	66.40%	2.99	^12^
	ZnCl_2_-activated carbon	Cu	90.00%	13.24	^12^
Agave agasse	Unmodified	Pb	99.40%	93.14	^13^
	Unmodified	Cd	90.94%	28.50	^13^
	Unmodified	Zn	80.79%	24.66	^13^
Rice straw	Biochar/nitration and nitro reduction	Cd	72.10%	72.50	^15^
Coconut coir	NaOH	Cu	92.19%	–	^16^
Coconut shell	Activated carbon/lanthanum	Cr	78.00%	0.01	^17^
Corncob	Xanthate	Pb	79.94%	124.84	^18^
	Silica/nanocomposite	Pb	95.50%	11.00	^19^
Corn husk leaves	Bismuthiol I	Hg	98.50%	726.57	^20^
Peanut hull	Iron chloride and hexamethylenediamine	Cr	–	142.86	^21^
Sawdust	Poly(*N*-acryloyl-l-histidine)	Pb	91.50%	241.80	^22^
Sawdust	Chitosan nanocomposite beads	Cu	86.20%	7.32	^23^
Sawdust	Chitosan nanocomposite beads	Ni	82.84%	6.92	^23^
Paddy husk	Biochar	Pb	–	14.20	^24^
	Biochar	Cu	–	10.27	^24^
	Biochar	Zn	–	6.48	^24^
Rice husk	Iron oxide	As	95.00%	82.00	^25^
	MgAl-layered double hydroxide	Cd	–	113.99	^26^
	MgAl-layered double hydroxide	Cu	–	101.29	^26^
Corncob	Aluminium-manganese	Cd	–	45.58	^27^
	Cerium oxide/nanocomposite	Cd	95.00%	–	^28^
	Cerium oxide/nanocomposite	Cr	88.00%	–	^28^
Oil palm bagasse	Al_2_O_3_ nanoparticles	Cd	87.00%	–	^29^
	Al_2_O_3_ nanoparticles	Ni	81.00%	–	^29^
Peanut shell	Fe_3_O_4_	Pb	–	188.68	^30^
*Tectona grandis* sawdust	Activated carbon modified with iron	As	75.00%	0.68	^31^
	Activated carbon modified with iron and zirconium	As	86.35%	1.21	^31^
*Lonicera japonica* flower	Biomass modified with iron oxide	Pb	–	14.49–23.26	^32^
		Cu	–	22.73–26.32	^32^
		Co	–	29.41–50.00	^32^

Many studies reported using various metal oxides of titanium dioxide (TiO_2_), zinc oxide (ZnO), aluminum oxide (Al_2_O_3_), magnesium oxide (MgO), and iron(II, III) oxide (Fe_3_O_4_ or Fe_2_O_3_) have been used to improve adsorbent efficiency by increasing surface area and adsorption capacity and giving fast reaction for heavy metal removals. In Table 1, various agriculture wastes modified with metal oxides have been used for removing heavy metals. For rice husk, the previous studies have been modified by iron oxide and MgAl-layered double hydroxide for removing arsenic (As), Cd(II), and Cu(II)^25,26^. Various modifications of corncob by aluminum-manganese and cerium oxide nanocomposite for Cd(II) and Cr(II) removals^27,28^. For oil palm bagasse, it has been modified by aluminum oxide (Al_2_O_3_) for eliminating Cd(II) and (Ni)^29^. In addition, the peanut shell was modified with iron(II, III) oxide (Fe_3_O_4_) for Pb(II) removal^30^. Sawdust is also used for producing activated carbon and modified with iron (Fe) and zirconium (Zr) for eliminating As(II)^31^. Furthermore, *Lonicera japonica* flower has been studied for producing biomass modified with iron oxide for Pb(II), Cu(II), and cobalt (Co) removals^32^. Among those metals, iron(III) oxide-hydroxide presented high adsorbent efficiency to deal high strength of heavy metals in wastewater^33^, so it is a good idea to use it to improve the efficiency of sugarcane bagasse for lead adsorption in an aqueous solution. In addition, the modification method of stable material is another point to consider for real industrial applications. However, no one modified sugarcane bagasse by changing material form or adding metal oxide, or both to improve material efficiency for lead adsorption in an aqueous solution. Therefore, this study attempts to synthesize sugarcane bagasse adsorbent materials modified with iron(III) oxide-hydroxide in powder and bead materials, compare their lead removal efficiencies through batch experiments, and verify whether adding metal oxide or changing form helped to improve material efficiency for lead adsorption.

The study aimed to synthesize four types of adsorbent materials which were sugarcane bagasse powder (SB), sugarcane bagasse powder doped iron(III) oxide-hydroxide (SBF), sugarcane bagasse beads (SBB), and sugarcane bagasse powder doped iron(III) oxide-hydroxide beads (SBFB). Several characterized techniques of X-Ray Diffractometer (XRD), Field Emission Scanning Electron Microscopy and Focus Ion Beam (FESEM-FIB) with Energy Dispersive X-Ray Spectrometer (EDX), and Fourier Transform Infrared Spectroscopy (FT-IR) were used to investigate their crystalline formations, surface morphologies, chemical compositions, and chemical functional groups. In addition, the point of zero charges (pH_pzc_) of all materials was studied to identify which pH value had the highest lead removal efficiency. Their lead removal efficiencies were also examined by batch experiments with varying doses, contact time, pH, and concentration. Moreover, their lead adsorption pattern and mechanisms were studied by linear and nonlinear adsorption isotherms of Langmuir, Freundlich, Temkin, and Dubinin–Radushkevich models and kinetics of pseudo-first-order, pseudo-second-order, elovich, and intraparticle diffusion models. Finally, the desorption experiments were investigated to confirm the reusability of the materials.

## Result and discussion

### The physical characteristics of sugarcane bagasse adsorbent materials

**Figure 1:**
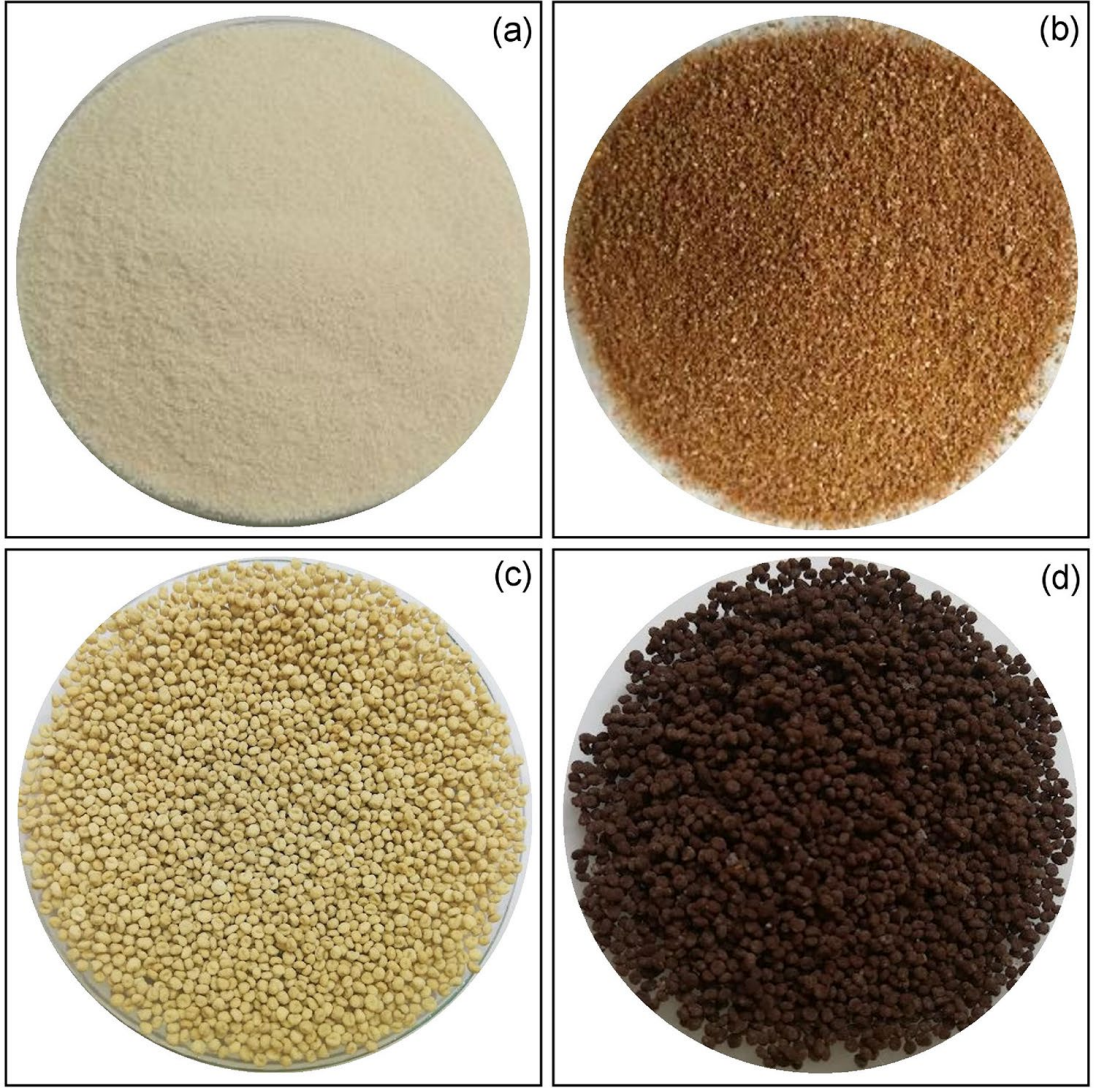
The physical characteristics of (**a**) sugarcane bagasse powder (SB), (**b**) sugarcane bagasse powder doped iron(III) oxide-hydroxide (SBF), (**c**) sugarcane bagasse powder beads (SBB), and (**d**) sugarcane bagasse powder doped iron(III) oxide-hydroxide beads (SBFB).

The physical characteristics of SB, SBF, SBB, and SBFB are demonstrated in Fig. 1a–d. For a powder form, SB was yellow color powder shown in Fig. 1a, whereas SBF was brown color powder shown in Fig. 1b. For a bead form, SBB was yellow color beads which had the same color as SB shown in Fig. 1c, whereas SBFB was iron-rust color beads which had a darker brown color than SBF shown in Fig. 1d.

### Characterizations of sugarcane bagasse adsorbent materials

#### The crystalline patterns of sugarcane bagasse adsorbent materials

**Figure 2:**
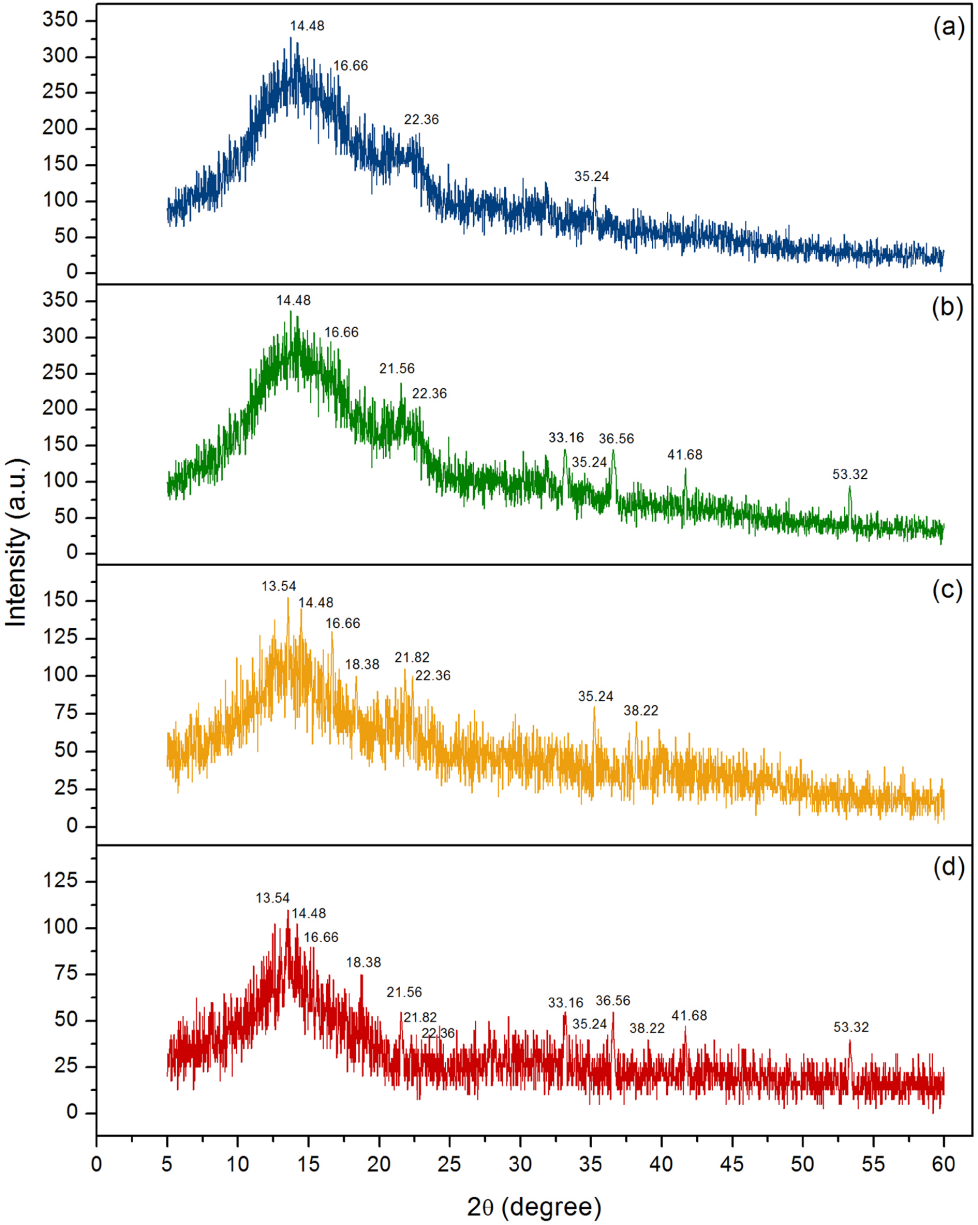
The crystalline patterns of (**a**) sugarcane bagasse powder (SB), (**b**) sugarcane bagasse powder doped iron(III) oxide-hydroxide (SBF), (**c**) sugarcane bagasse powder beads (SBB), and (**d**) sugarcane bagasse powder doped iron(III) oxide-hydroxide beads (SBFB) by XRD analysis.

The crystalline formations of SB, SBF, SBB, and SBFB were determined by XRD analysis, and their XRD patterns are presented in Fig. 2a–d. They were amorphous phases with presenting specific peaks of cellulose at 14.48°, 16.66°, 22.36°, and 35.24°^34^. The sodium alginate peaks of 13.54°, 18.38°, 21.82°, and 38.22°^35^ were detected in SBB and SBFB shown in Fig. 2c,d. In addition, the specific peaks of iron(III) oxide-hydroxide peaks of 21.56°, 33.16°, 36.56°, 41.68°, and 53.32° matched to JCPDS no. 29-0713^36^ were only found in SBF and SBFB showed in Fig. 2b,d.

#### The surface morphologies of sugarcane bagasse adsorbent materials

**Figure 3:**
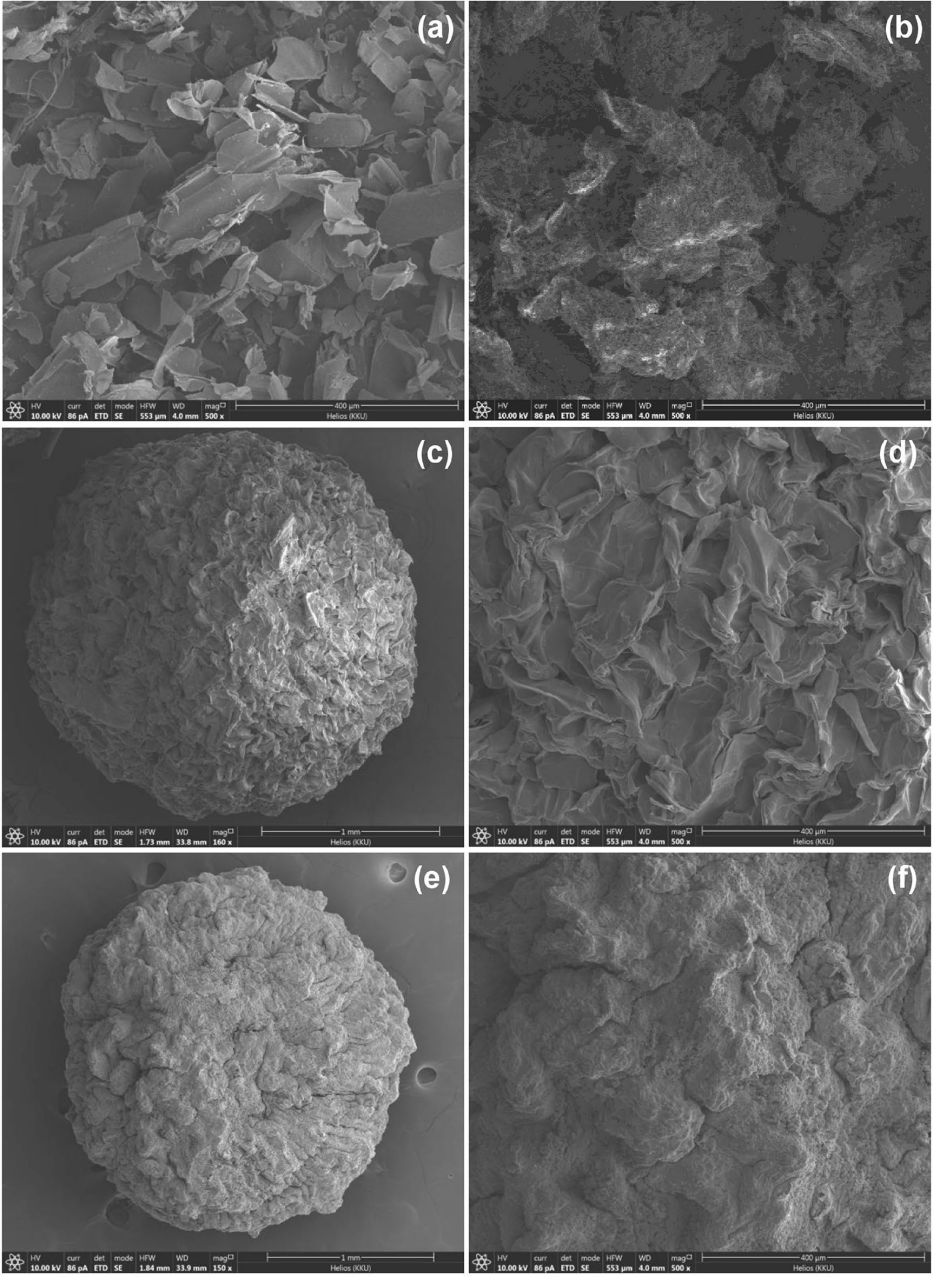
The surface morphologies of (**a**) sugarcane bagasse powder (SB), (**b**) sugarcane bagasse powder doped iron(III) oxide-hydroxide (SBF), (**c**,**d**) sugarcane bagasse powder beads (SBB) in bead and surface, and (e and f) sugarcane bagasse powder doped iron(III) oxide-hydroxide beads (SBFB) in bead and surface by FESEM-FIB analysis.

The surface morphologies of SB, SBF, SBB, and SBFB by FESEM-FIB analysis at 500X magnification with 400 µm for a surface and at 150-160X magnification with 1 mm for a bead illustrated in Fig. 3a–f. For SB, its surface was scales or overlapping plates shown in Fig. 3a. For SBF, its surface was similar to SB with having an iron(III) oxide-hydroxide in a hairy rod shape covered on the surface as shown in Fig. 3b. For SBB, it had a spherical shape with a coarse surface at 160X magnification with 1 mm. Its surface was scaly when was zoomed at 500X magnification with 400 µm demonstrated in Fig. 3c,d. Finally, SBFB had a spherical shape with a coarse surface at 150× magnification of 1 mm. Its surface was heterogenous and coarse surface when zoomed at 500× magnification with 400 µm illustrated in Fig. 3e,f.

#### The chemical functional compositions of sugarcane bagasse adsorbent materials

The chemical compositions of SB, SBF, SBB, and SBFB were analyzed by using EDX analysis represented in Table 2. Three main chemical components of carbon (C), oxygen (O), and calcium (Ca) were found in all sugarcane bagasse adsorbent materials. For sodium (Na) and chloride (Cl), they were detected in all sugarcane bagasse adsorbent materials except SB. For iron (Fe), it was observed only in SBF and SBFB with the addition of iron(III) oxide-hydroxide. For SB and SBF, the mass percentages by weight of C and O were decreased when iron(III) oxide-hydroxide was added to SB, whereas the mass percentage by weight of Ca, Na, Cl, and Fe were increased. The increase in those chemical compositions might be from using chemicals in a process of adding iron(III) oxide-hydroxide by ferric chloride hexahydrate (FeCl_3_·6H_2_O) and sodium hydroxide (NaOH) similar to a previous study reported^37^. For SB and SBB, the mass percentage by weight of C and O decreased when it was changed a form from powder form to bead form, whereas the mass percentage by weight of Ca, Na, and Cl were increased. The increase in those chemical compositions might be from using chemicals in a process of bead formation by sodium alginate (NaC_6_H_7_O_6_) and calcium chloride (CaCl_2_) supported by the previous studies on the effect of bead formation of materials^38–41^. For SBF and SBFB, the mass percentage by weight of C, O, and Fe were decreased when SBF was changed to bead form which the decreasing of Fe content might be interfered from the bead formation process. While the mass percentage by weight of Ca, Na, and Cl were increased which might be from using chemicals of NaC_6_H_7_O_6_ and CaCl_2_ in a process of bead formation similar to SBB. Therefore, the addition of iron(III) oxide-hydroxide and changing the material form affected the increase of Ca, Na, Cl, and Fe contents in sugarcane bagasse adsorbent materials.

**Table 2:** The chemical compositions of sugarcane bagasse powder (SB), sugarcane bagasse powder doped iron(III) oxide-hydroxide (SBF), sugarcane bagasse powder beads (SBB), and sugarcane bagasse powder doped iron(III) oxide-hydroxide beads (SBFB) in the percentages by weight.

Chemical compositions (%wt)	Adsorbent materials			
	SB	SBF	SBB	SBFB
C	51.2	41.6	44.7	39.7
O	46.4	44.5	45.8	40.1
Ca	2.4	2.5	8.4	8.6
Na	–	2.1	0.5	2.5
Cl	–	3.0	0.6	3.5
Fe	–	6.3	–	5.6

#### The chemical functional groups of sugarcane bagasse adsorbent materials

**Figure 4:**
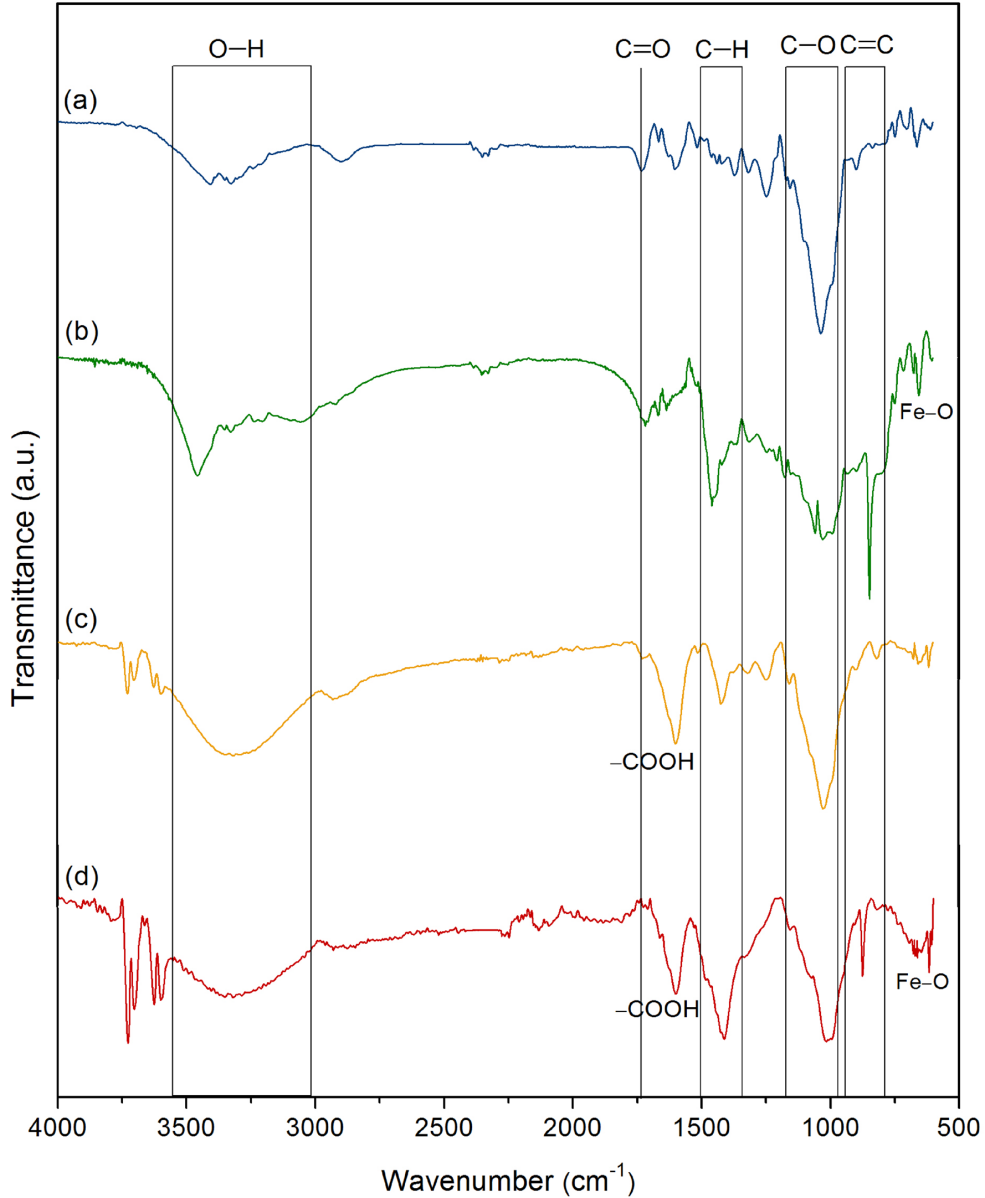
The chemical functional groups of (**a**) sugarcane bagasse powder (SB), (**b**) sugarcane bagasse powder doped iron(III) oxide-hydroxide (SBF), (**c**) sugarcane bagasse powder beads (SBB), and (**d**) sugarcane bagasse powder doped iron(III) oxide-hydroxide beads (SBFB) by FT-IR spectra analysis.

The chemical functional groups of SB, SBF, SBB, and SBFB were examined by FT-IR analysis, and their FT-IR spectra are demonstrated in Fig. 4a–d. Five main chemical function groups of O–H, C=O, C–H, C–O, and C=C were observed in all materials. The carboxyl group (–COOH) of sodium alginate was only detected in bead materials (SBB and SBFB), and Fe–O was only found in materials with adding iron(III) oxide-hydroxide (SBF and SBFB)^34,42^. For O–H, it represented the stretching of hydroxyl and phenolic groups, and C=O referred to the stretching of the carbonyl bond of hemicellulose and lignin aromatic group in the sugarcane bagasse^43^. C–H was the stretching of the CH_2_ group of cellulose, and C–O was the stretching of alcohol and carboxylic acid^44^. Finally, C=C was the stretching of hemicellulose and cellulose in the sugarcane bagasse^34^. For SB, the peaks at 3349.84, 1730.39, 1438.70, 1035.43, and 897.32 cm^−1^ indicated the stretching of O–H, C=O, C–H, C–O, and C=C, respectively shown in Fig. 4a. For SBF, the peaks at 3350.74, 1716.81, 1457.49, 1057.54, 847.21, and 604.93 cm^−1^ represented the stretching of O–H, C=O, C–H, C–O, C=C, and Fe–O, respectively demonstrated in Fig. 4b. For SBB, the peaks at 3316.75, 1737.80, 1591.22, 1421.99, 1026.45, and 883.37 cm^−1^ were the stretching of O–H, C=O, –COOH, C–H, C–O, and C=C, respectively illustrated in Fig. 4c. For SBFB, the peaks at 3320.20, 1728.16, 1556.50, 1412.34, 1016.74, 875.11, and 617.20 cm^−1^ referred the stretching of O–H, C=O, –COOH, C–H, C–O, C=C, and Fe–O, respectively shown in Fig. 4d.

#### The point of zero charges of sugarcane bagasse adsorbent materials

**Figure 5:**
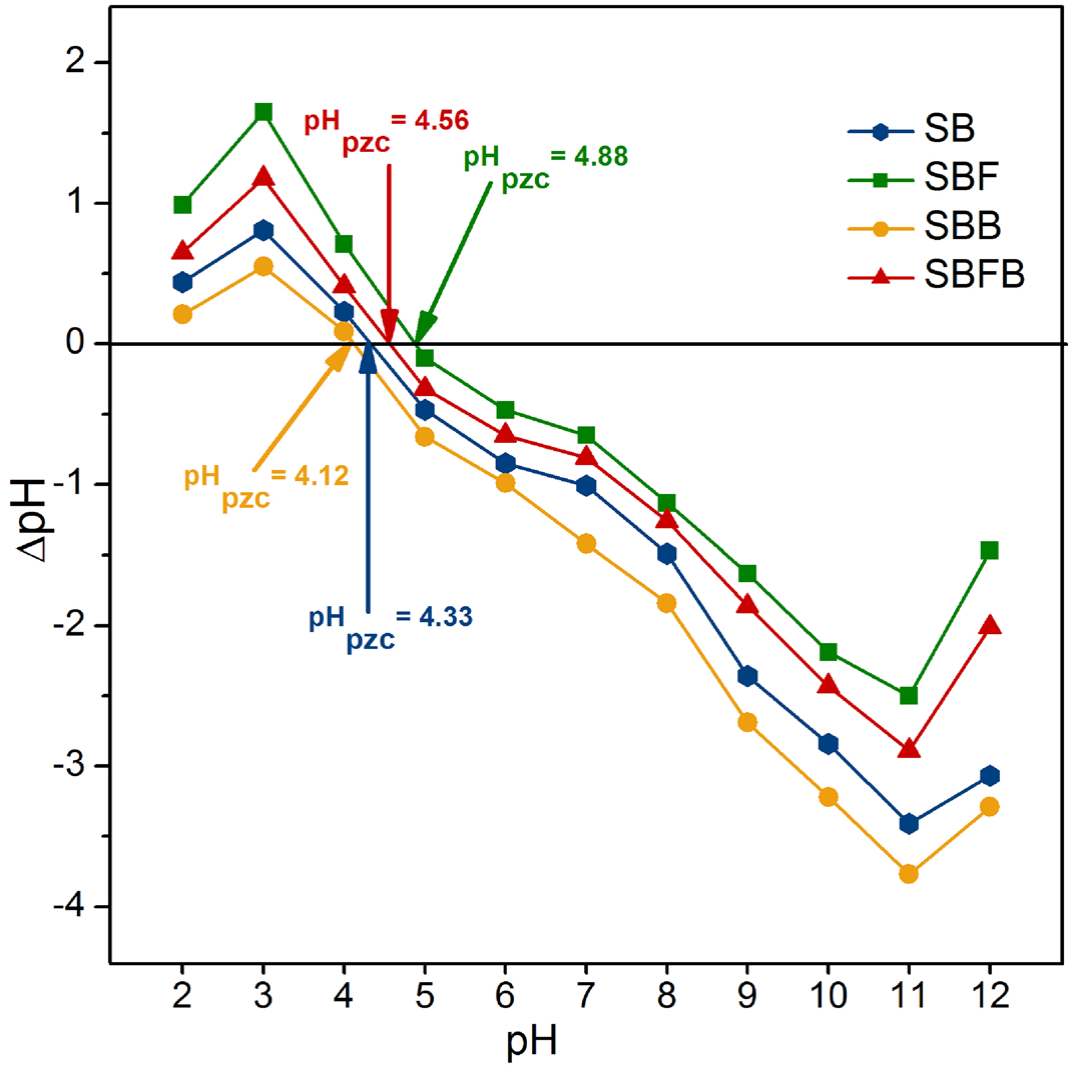
The point of zero charges of sugarcane bagasse powder (SB), sugarcane bagasse powder doped iron(III) oxide-hydroxide (SBF), sugarcane bagasse powder beads (SBB), and sugarcane bagasse powder doped iron(III) oxide-hydroxide beads (SBFB) for lead adsorptions.

The point of zero charges (pH_pzc_) of sugarcane bagasse adsorbent materials was studied for determining which pH value of each material is good for lead adsorption. In theory, a pH value that gives the net charge equal to zero is a pH_pzc_ of the adsorbent^41^. The pH_pzc_ of SB, SBF, SBB, and SBFB are demonstrated in Fig. 5. The pH_pzc_ of SB, SBF, SBB, and SBFB were 4.33, 4.88, 4.12, and 4.56, respectively, so the addition of iron(III) oxide-hydroxide or the changing material form affected to the increase of pH_pzc_. The high lead adsorption should happen at the pH of solution higher than the pH_pzc_ (pH_solution_ > pH_pzc_) of adsorbent because its surface is negatively charged^33,45^. Therefore, the high lead removal efficiencies of sugarcane adsorbent materials in this study should be found at pH > 4.

### Batch adsorption experiments

#### The effect of dose

**Figure 6:**
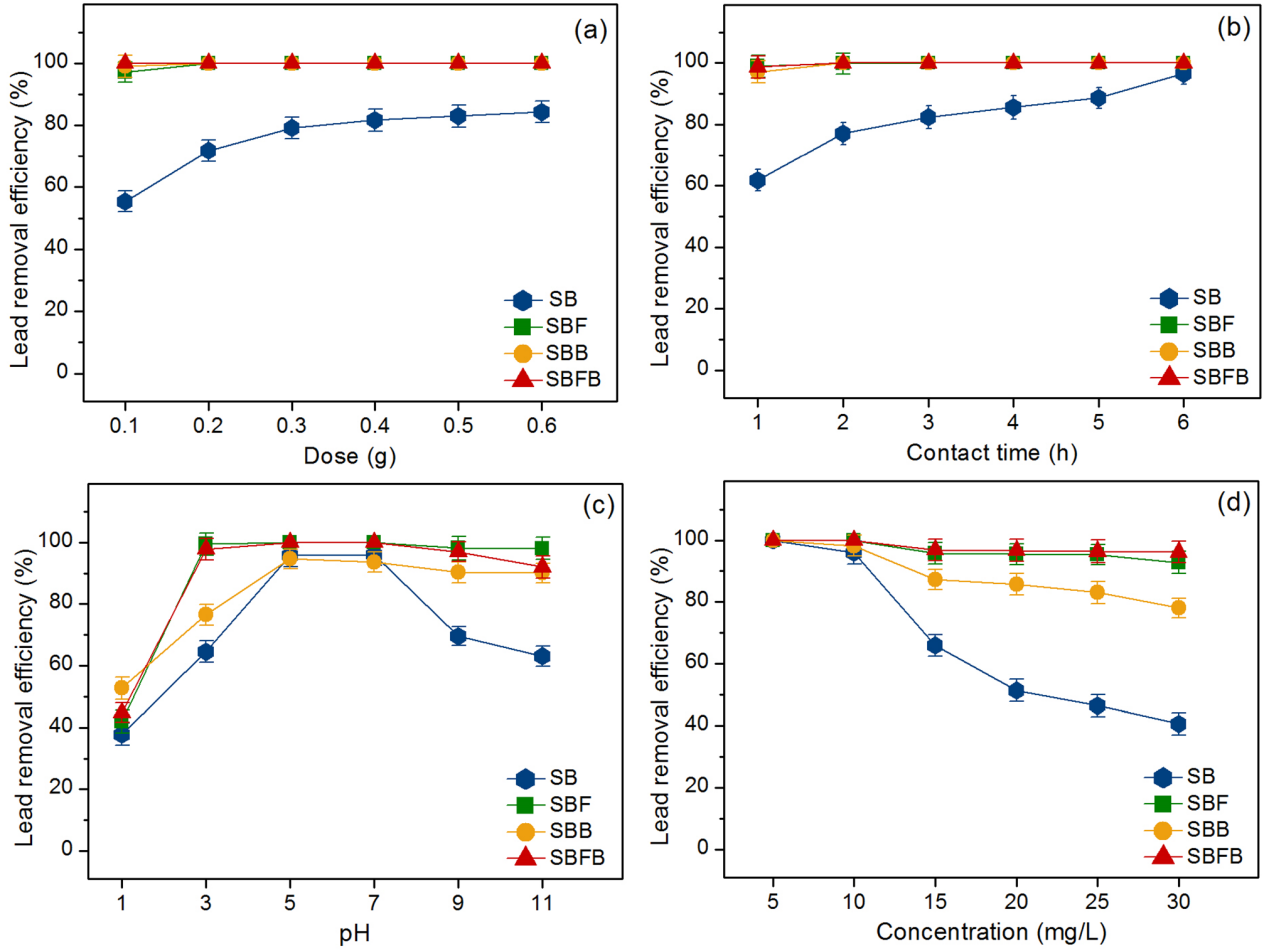
Batch experiments on the effects of (**a**) dose, (**b**) contact time, (**c**) pH, and (**d**) concentration of sugarcane bagasse powder (SB), sugarcane bagasse powder doped iron(III) oxide-hydroxide (SBF), sugarcane bagasse powder beads (SBB), and sugarcane bagasse powder doped iron(III) oxide-hydroxide beads (SBFB).

Six different doses from 0.1 to 0.6 g were used for investigating the dose effect of lead adsorptions by sugarcane bagasse adsorbent materials, and the results are demonstrated in Fig. 6a. The control condition was the lead concentration of 10 mg/L, a sample volume of 200 mL, a contact time of 4 h, pH 5, a temperature of 25 °C, and a shaking speed of 200 rpm. In Fig. 6a, lead removal efficiencies of all materials were increased with the increase of material dose which might be from the increase of surface area or active sites of materials similarly found in previous studies^33,37,38,45^. The highest lead removal efficiency of SB was 84.29% at 0.6 g whereas the highest lead removal efficiencies of SBF, SBB, and SBFB were 100% at 0.2 g, 0.2 g, and 0.1 g, respectively. Therefore, these were found as the optimum dose of sugarcane bagasse adsorbent materials that were used for studying the contact time effect.

#### The effect of contact time

The different contact times from 1 to 6 h were used for studying the contact time effect on lead adsorptions by sugarcane bagasse adsorbent materials, and the results are demonstrated in Fig. 6b. The control condition was the lead concentration of 10 mg/L, a sample volume of 200 mL, pH 5, a temperature of 25 °C, a shaking speed of 200 rpm, and the optimum dose of 0.6 g (SB) or 0.2 g (SBF and SBB) or 0.1 g (SBFB). In Fig. 6b, lead removal efficiencies of all materials were increased with the increase of contact time similar to the dose effect. The highest lead removal efficiency represented the saturated lead adsorption on bagasse adsorbent materials. In Fig. 6b, the highest lead removal efficiency of SB was 96.49% at 6 h whereas the highest lead removal efficiencies of SBF, SBB, and SBFB were 100% at 3 h, 2 h, and 2 h, respectively. Therefore, these were the optimum contact time of sugarcane bagasse adsorbent materials that were used for studying the pH effect.

#### The effect of pH

The effect of pH was studied by varying pH values of 1, 3, 5, 7, 9, and 11 which represented the acid, neutral, and alkaline conditions on lead adsorptions by sugarcane bagasse adsorbent materials, and the results are demonstrated in Fig. 6c. The control condition was the lead concentration of 10 mg/L, a sample volume of 200 mL, a temperature of 25 °C, a shaking speed of 200 rpm, and the optimum dose of 0.6 g (SB) or 0.2 g (SBF and SBB) or 0.1 g (SBFB) and contact time at 6 h (SB) or 3 h (SBF) or 2 h (SBB and SBFB). In Fig. 6c, lead removal efficiencies of all materials were increased with the increase of pH values from 1 to 7, then they were decreased. The highest lead removal efficiencies of all materials were found at pH 5 with lead removal at 95.88%, 100%, 94.66%, and 100% for SB, SBF, SBB, and SBFB, respectively. This result agreed with the pH_pzc_ results of this study which the pH_pzc_ values of all materials were found at pH > 4. Moreover, it also corresponded to other previous studies that reported the highest lead removal efficiency at pH > 4 relating to pH_pzc_ of lead adsorptions in an aqueous solution^33,37,45,46^. Therefore, pH 5 was the optimum pH of all sugarcane bagasse adsorbent materials which were used for studying the concentration effect.

#### The effect of concentration

Several lead concentrations of 5–30 mg/L were used to investigate the concentration effect on lead adsorptions by sugarcane bagasse adsorbent materials, and the results are demonstrated in Fig. 6d. The control condition was a sample volume of 200 mL, a temperature of 25 °C, a shaking speed of 200 rpm, and the optimum dose of 0.6 g (SB) or 0.2 g (SBF and SBB) or 0.1 g (SBFB), contact time at 6 h (SB) or 3 h (SBF) or 2 h (SBB and SBFB), and pH of 5. In Fig. 6d, lead removal efficiencies of all materials were decreased with the increase of concentration because lead ions were more than the available active sites of bagasse adsorbent materials similar to the report by other studies^9,33,37,38,45^. Lead removal efficiencies from 5 to 30 mg/L of SB, SBF, SBB, and SBFB were 40.63–100%, 92.78–100%, 78.07–100%, and 96.19–100%, respectively. For the lead concentration of 10 mg/L, lead removal efficiencies of SB, SBF, SBB, and SBFB were 96.08%, 100%, 98.22%, and 100%, respectively, and SBFB demonstrated the highest lead removal efficiency of other materials.

In conclusion, 0.6 g, 6 h, pH 5, 10 mg/L, 0.2 g, 3 h, pH 5, 10 mg/L, 0.2 g, 2 h, pH 5, 10 mg/L, and 0.1 g, 2 h, pH 5, 10 mg/L were the optimum conditions in dose, contact time, pH, and concentration of SB, SBF, SBB, and SBFB, respectively, and they could be arranged in order from high to low of SBFB > SBF > SBB > SB. As a result, the changing material form and adding iron(III) oxide-hydroxide helped to improve material efficiency for lead adsorption, and SBFB was recommended to be further applied for lead removal in the wastewater treatment system.

### Adsorption isotherms

**Figure 7:**
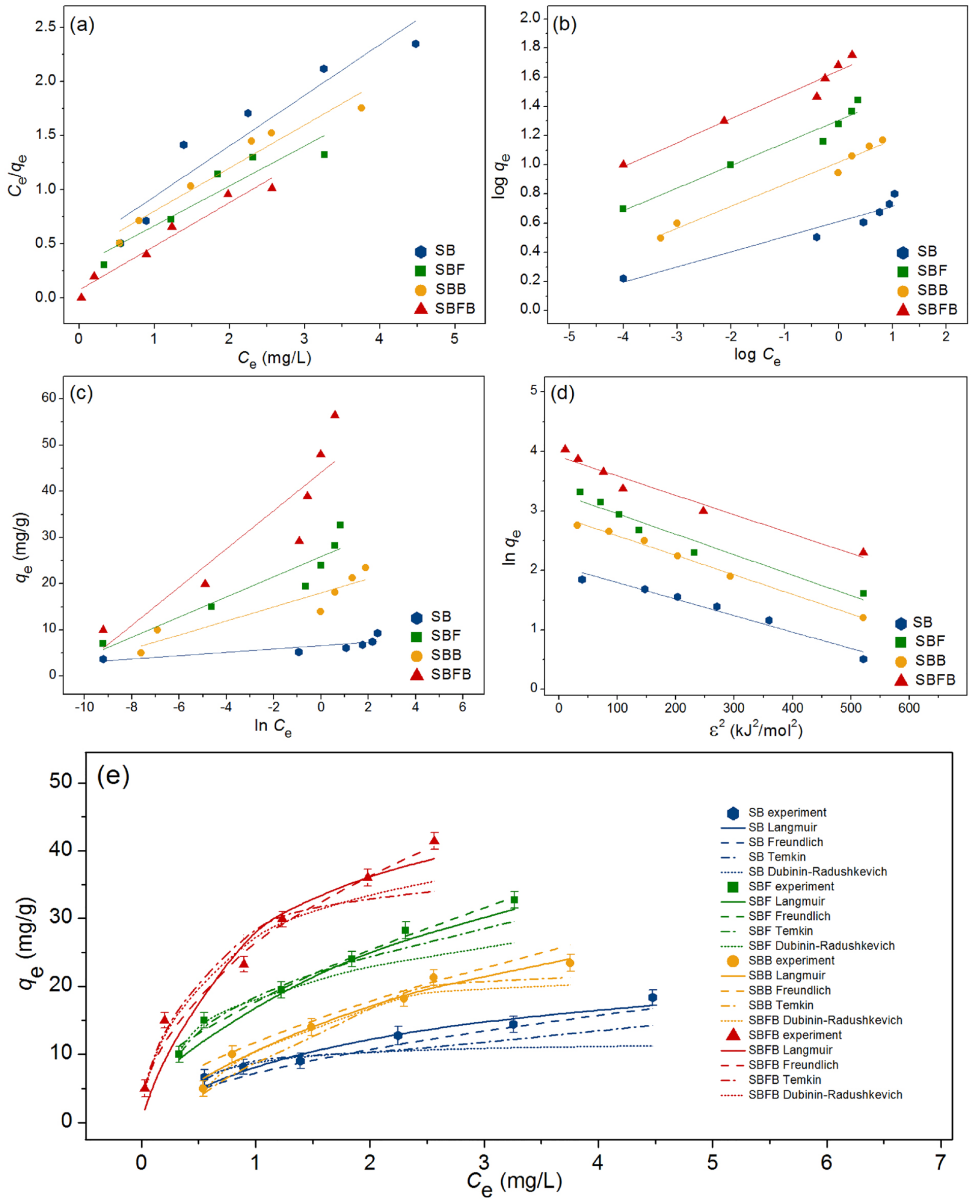
Graphs of (**a**) linear Langmuir, (**b**) linear Freundlich, (**c**) linear Temkin, (**d**) linear Dubinin–Radushkevich, and (**e**) nonlinear adsorption isotherms of sugarcane bagasse powder (SB), sugarcane bagasse powder doped iron(III) oxide-hydroxide (SBF), sugarcane bagasse powder beads (SBB), and sugarcane bagasse powder doped iron(III) oxide-hydroxide beads (SBFB) for lead adsorptions.

The adsorption isotherm was designed to investigate the adsorption patterns of SB, SBF, SBB, and SBFB for lead adsorptions. Linear and nonlinear models of Langmuir, Freundlich, Temkin, and Dubinin–Radushkevich models were studied. For linear models, Langmuir, Freundlich, Temkin, and Dubinin–Radushkevich isotherms were plotted by *C*_e_/*q*_e_ versus *C*_e_, log *q*_e_ versus log *C*_e_, *q*_e_ versus ln *C*_e_, and ln *q*_e_ versus *ε*^2^, respectively. For nonlinear models, all isotherms were plotted by* C*_e_ versus *q*_e._ The graph plotting results and the equilibrium isotherm parameters were demonstrated in Fig. 7a–e and Table 3, respectively.

**Table 3:** The comparison of linear and nonlinear isotherm parameters for lead adsorptions on sugarcane bagasse powder (SB), sugarcane bagasse powder doped iron(III) oxide-hydroxide (SBF), sugarcane bagasse powder beads (SBB), and sugarcane bagasse powder doped iron(III) oxide-hydroxide beads (SBFB).

Regression methods	Isotherm models	Parameters	SB	SBF	SBB	SBFB
Linear	Langmuir	*q*_m_ (mg/g)	6.161	27.027	23.697	57.471
		*K*_L_ (L/mg)	1.337	5.362	3.431	6.692
		*R*^2^	0.966	0.944	0.987	0.948
	Freundlich	1*/n*	0.104	0.155	0.125	0.164
		*K*_F_ (mg/g) (L/mg)^1/n^	4.091	20.160	17.022	44.005
		*R*^2^	0.930	0.951	0.861	0.951
	Temkin	*b*_T_ (× 10^3^) (J/mol)	7.457	1.295	1.629	0.599
		*A*_T_ (× 10^5^) (L/g)	5.427	0.510	1.403	0.429
		*R*^2^	0.790	0.813	0.867	0.818
	Dubinin–Radushkevich	*q*_*m*_ (mg/g)	4.648	19.556	19.184	40.419
		*K*_DR_ (mol^2^/J^2^)	0.002	0.003	0.003	0.003
		*E* (kJ/mol)	15.811	13.363	12.500	13.363
		*R*^2^	0.769	0.832	0.838	0.843
Nonlinear	Langmuir	*q*_m_ (mg/g)	6.193	28.027	23.659	57.505
		*K*_L_ (L/mg)	1.357	5.452	3.420	6.651
		*R*^2^	0.968	0.946	0.985	0.951
		*R*_adj_^2^	0.960	0.946	0.982	0.950
		RMSE	0.383	5.493	5.576	10.787
	Freundlich	1*/n*	0.105	0.154	0.127	0.167
		*K*_F_ (mg/g) (L/mg)^1/n^	4.139	20.165	17.019	45.029
		*R*^2^	0.931	0.953	0.866	0.955
		*R*_adj_^2^	0.913	0.952	0.860	0.954
		RMSE	0.383	2.909	2.438	5.640
	Temkin	*b*_T_ (× 10^3^) (J/mol)	7.461	1.298	1.626	0.609
		*A*_T_ (L/g)	5.467	0.516	1.415	0.433
		*R*^2^	0.791	0.811	0.865	0.819
		*R*_adj_^2^	0.788	0.810	0.863	0.817
		RMSE	0.850	4.099	2.874	8.310
	Dubinin–Radushkevich	*q*_*m*_ (mg/g)	4.660	19.560	19.176	41.190
		*K*_DR_ (mol^2^/J^2^)	0.002	0.003	0.005	0.004
		*E* (kJ/mol)	15.233	12.467	10.266	11.924
		*R*^2^	0.768	0.831	0.831	0.845
		*R*_adj_^2^	0.766	0.829	0.829	0.844
		RMSE	0.680	3.635	1.962	7.402

For the linear Langmuir model, Langmuir maximum adsorption capacities (*q*_m_) of SB, SBF, SBB, and SBFB were 6.161, 27.027, 23.697, and 57.471 mg/g, respectively, and Langmuir adsorption constants (*K*_L_) of SB, SBF, SBB, and SBFB were 1.337, 5.362, 3.431, and 6.692 L/mg, respectively. For the nonlinear Langmuir model, Langmuir maximum adsorption capacities (*q*_m_) of SB, SBF, SBB, and SBFB were 6.193, 28.027, 23.659, and 57.505 mg/g, respectively, and Langmuir adsorption constants (*K*_L_) of SB, SBF, SBB, and SBFB were 1.357, 5.452, 3.420, and 6.651 L/mg, respectively. For the linear Freundlich isotherm, the 1/*n* values of SB, SBF, SBB, and SBFB were 0.104, 0.155, 0.125, and 0.164, respectively. Freundlich adsorption constants (*K*_F_) of SB, SBF, SBB, and SBFB were 4.091, 20.160, 17.022, and 44.005 (mg/g) (L/mg)^1/n^, respectively. For the nonlinear Freundlich isotherm, the 1/*n* values of SB, SBF, SBB, and SBFB were 0.105, 0.154, 0.127, and 0.167, respectively. Freundlich adsorption constants (*K*_F_) of SB, SBF, SBB, and SBFB were 4.139, 20.165, 17.019, and 45.029 (mg/g) (L/mg)^1/n^, respectively. For the linear Temkin isotherm, *b*_T_ values of SB, SBF, SBB, and SBFB were 7.457 × 10^3^, 1.295 × 10^3^, 1.629 × 10^3^, and 0.599 × 10^3^ J/mol, respectively. *A*_T_ values of SB, SBF, SBB, and SBFB were 5.427 × 10^5^, 0.510 × 10^5^, 1.403 × 10^5^, and 0.429 × 10^5^ L/g, respectively. For the nonlinear Temkin isotherm, *b*_T_ values of SB, SBF, SBB, and SBFB were 7.461 × 10^3^, 1.298 × 10^3^, 1.626 × 10^3^, and 0.609 × 10^3^ J/mol, respectively. *A*_T_ values of SB, SBF, SBB, and SBFB were 5.467 × 10^5^, 0.516 × 10^5^, 1.415 × 10^5^, and 0.433 × 10^5^ L/g, respectively. For the linear Dubinin–Radushkevich model, the maximum adsorption capacities (*q*_m_) of SB, SBF, SBB, and SBFB were 4.648, 19.556, 19.184, and 40.419 mg/g, respectively, and the activity coefficient (*K*_DR_) values of SB, SBF, SBB, and SBFB were 0.002, 0.003, 0.003, and 0.003 mol^2^/J^2^, respectively. The adsorption energy (*E*) values of SB, SBF, SBB, and SBFB were 15.811, 13.363, 12.500, and 13.363 kJ/mol, respectively. For the nonlinear Dubinin–Radushkevich model, the maximum adsorption capacities (*q*_m_) of SB, SBF, SBB, and SBFB were 4.660, 19.560, 19.176, and 40.190 mg/g, respectively, and the activity coefficient (*K*_DR_) values of SB, SBF, SBB, and SBFB were 0.002, 0.003, 0.005, and 0.004 mol^2^/J^2^, respectively. The adsorption energy (*E*) values of SB, SBF, SBB, and SBFB were 15.233, 12.467, 10.266, and 11.924 kJ/mol, respectively.

For the *R*^2^ value consideration, the *R*^2^ values of SB, SBF, SBB, and SBFB in the linear Langmuir model were 0.966, 0.944, 0.987, and 0.948, respectively, and their *R*^2^ values in the linear Freundlich model were 0.930, 0.951, 0.861, and 0.951, respectively. For the linear Temkin model, the *R*^2^ values of SB, SBF, SBB, and SBFB were 0.790, 0.813, 0.867, and 0.818, respectively, and their *R*^2^ values in the linear Dubinin–Radushkevich were 0.769, 0.832, 0.838, and 0.843, respectively. In addition, the *R*^2^ values of SB, SBF, SBB, and SBFB in the nonlinear Langmuir model were 0.968, 0.946, 0.985, and 0.951, respectively, and their *R*^2^ values in the nonlinear Freundlich model were 0.931, 0.953, 0.866, and 0.955, respectively. For the nonlinear Temkin model, the *R*^2^ values of SB, SBF, SBB, and SBFB were 0.791, 0.811, 0.865, and 0.819, respectively, and their *R*^2^ values in the nonlinear Dubinin–Radushkevich were 0.768, 0.831, 0.831, and 0.845, respectively. Moreover, the *R*_adj_^2^ values of SB, SBF, SBB, and SBFB in the nonlinear Langmuir model were 0.960, 0.946, 0.982, and 0.950, respectively, and their *R*_adj_^2^ values in the nonlinear Freundlich model were 0.913, 0.952, 0.860, and 0.954, respectively. the *R*_adj_^2^ values of SB, SBF, SBB, and SBFB in the nonlinear Temkin model were 0.788, 0.810, 0.863, and 0.817, respectively, and their *R*_adj_^2^ values in nonlinear Dubinin–Radushkevich were 0.766, 0.829, 0.829, and 0.844.

Since the *R*^2^ values of SB and SBB in both linear and nonlinear Langmuir models were higher than Freundlich, Temkin, and Dubinin–Radushkevich models, their adsorption patterns corresponded to Langmuir isotherm relating to a physical process with homogenous adsorption on adsorbent surfaces. Therefore, the maximum adsorption capacity (*q*_m_) and Langmuir adsorption constant (*K*_L_) were used for considering lead adsorption by SB and SBB. In Table 3, SBB demonstrated higher *q*_m_ and *K*_L_ values than SB, so SBB had higher lead adsorption with a faster reaction than SB. While the *R*^2^ values of SBF and SBFB in both linear and nonlinear Freundlich models were higher than Langmuir, Temkin, and Dubinin–Radushkevich models, their adsorption patterns were Freundlich isotherm relating to a physiochemical process with heterogeneous adsorption on adsorbent surfaces. Therefore, the Freundlich adsorption constant (*K*_F_) and a constant depiction of adsorption intensity (1/*n*) were used for explaining lead adsorption by SBF and SBFB. In Table 3, SBFB illustrated a higher *K*_F_ value than SBF, so SBFB might be faster lead adsorption than SBF. Since 1/*n* values of SBF and SBFB had less than 1 that meant both materials were favorable lead adsorptions. Moreover, both linear and nonlinear isotherm models were recommended to plot graphs for confirming the results and protecting against data mistranslation^45,47–50^.

Moreover, the comparison of the maximum adsorption capacity (*q*_m_) value of sugarcane bagasse adsorbent materials with and without modifications for lead adsorption in an aqueous solution is illustrated in Table 4. For unmodified comparison, SB had a higher *q*_m_ value than the study of Ezeonuegbu et al.^9^ while it had a closely *q*_m_ value to Matin-Lara et al.^51^. For modified comparison, the *q*_m_ values of SB, SBF, SBB, and SBFB had higher values than other studies except for the studies of modified sugarcane bagasse with acid, alkaline, or metal oxides with acid or alkaline modifications. In this study, SBFB presented the highest *q*_m_ value which meant that SBFB had possibly the highest lead adsorption property than other materials.

**Table 4:** Comparison of the maximum adsorption capacity for lead adsorption of various sugarcane bagasse adsorbents.

Modifications	*q*_m_ (mg/g)	Refs
Unmodified	1.61	^9^
Unmodified	6.37	^51^
Biochar	12.74	^10^
Citric acid	52.63	^52^
Sulphuric acid	7.30	^51^
Nitric acid	18.72	^14^
Sodium hydroxide	21.24	^14^
Succinic anhydride	416.70	^46^
Succinic anhydride/alginate	354.60	^34^
Hydrothermal carbonization (HTC)/KOH activation	92.24	^53^
Fe_3_O_4_/citric acid	116.70	^54^
Fe_3_O_4_/co-precipitation	526.32	^55^
Fe_3_O_4_/formaldehyde	555.56	^55^
Fe_3_O_4_/NaOH and urea	666.67	^55^
Nano-hematite	16.57	^42^
ZnCl_2_-activated carbon	19.30	^12^
Sugarcane bagasse powder (SB)	6.16	This study
Sugarcane bagasse powder doped iron(III) oxide-hydroxide (SBF)	27.03	This study
Sugarcane bagasse beads (SBB)	23.70	This study
Sugarcane bagasse powder doped iron(III) oxide-hydroxide beads (SBFB)	57.47	This study

### Adsorption kinetics

**Figure 8:**
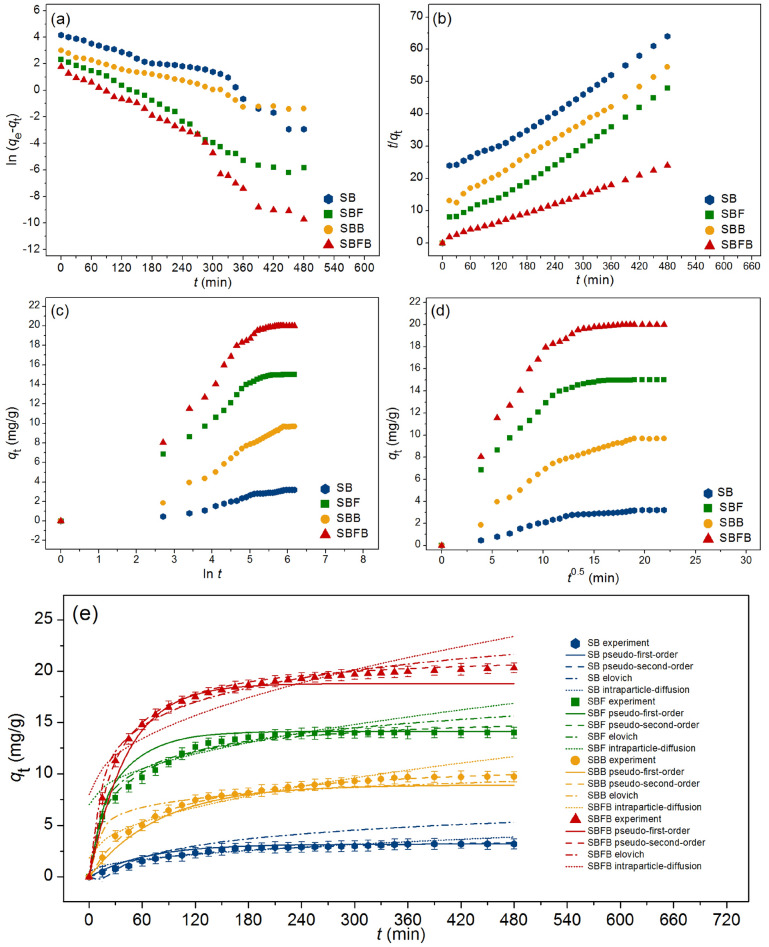
Graphs of (**a**) linear pseudo-first-order, (**b**) linear pseudo-second-order, (**c**) linear elovich model (**d**) linear intraparticle diffusion, and (**e**) nonlinear kinetic models of sugarcane bagasse powder (SB), sugarcane bagasse powder doped iron(III) oxide-hydroxide (SBF), sugarcane bagasse powder beads (SBB), and sugarcane bagasse powder doped iron(III) oxide-hydroxide beads (SBFB) for lead adsorptions.

The adsorption kinetics are designed for understanding the adsorption mechanism and rate of reaction of lead adsorptions by SB, SBF, SBB, and SBFB. Various kinetic models of the pseudo-first-order kinetic, pseudo-second-order kinetic, elovich, and intraparticle diffusion models for plotting linear and nonlinear models were investigated. For linear models, they were plotted by ln (*q*_e_ − *q*_t_) versus time (*t*), *t*/*q*_t_ versus time (*t*), *q*_t_ versus ln *t*, and *q*_t_ versus time (*t*^0.5^) for pseudo-first-order kinetic, pseudo-second-order kinetic, elovich, and intraparticle diffusion models, respectively. For nonlinear models, they were plotted by *q*_t_ versus time (*t*). The graph plotting results and the adsorption kinetic parameters were demonstrated in Fig. 8a–e and Table 5, respectively.

**Table 5:** The comparison of linear and nonlinear kinetic parameters for lead adsorptions on sugarcane bagasse powder (SB), sugarcane bagasse powder doped iron(III) oxide-hydroxide (SBF), sugarcane bagasse powder beads (SBB), and sugarcane bagasse powder doped iron(III) oxide-hydroxide beads (SBFB).

Regression methods	Kinetic models	Parameters	SB	SBF	SBB	SBFB
Linear	Pseudo-first-order kinetic	*q*_e_ (mg/g)	5.204	13.722	8.549	34.460
		*k*_1_ (min^−1^)	0.014	0.020	0.010	0.025
		*R*^2^	0.911	0.979	0.975	0.968
	Pseudo-second-order kinetic	*q*_e_ (mg/g)	3.934	11.455	10.965	21.053
		*k*_2_ (g/mg min)	0.003	0.002	0.002	0.003
		*R*^2^	0.991	0.992	0.992	0.998
	Elovich	*α* (mg/g/min)	0.047	3.334	4.354	0.918
		*β* (g/mg)	1.510	0.494	0.529	0.289
		*R*^2^	0.901	0.912	0.946	0.954
	Intraparticle diffusion	*k*_i_ (mg/g min^0.5^)	0.146	0.444	0.430	0.704
		*C*_i_ (mg/g)	0.490	2.276	1.756	7.974
		*R*^2^	0.867	0.814	0.906	0.730
Nonlinear	Pseudo-first-order kinetic	*q*_e_ (mg/g)	5.616	14.806	8.915	34.595
		*k*_1_ (min^−1^)	0.016	0.025	0.013	0.024
		*R*^2^	0.913	0.977	0.977	0.971
		*R*_adj_^2^	0.910	0.976	0.976	0.970
		RMSE	0.267	1.016	0.576	1.120
	Pseudo-second-order kinetic	*q*_e_ (mg/g)	3.946	11.903	11.300	21.471
		*k*_2_ (g/mg min)	0.003	0.001	0.001	0.002
		*R*^2^	0.993	0.995	0.996	0.996
		*R*_adj_^2^	0.992	0.994	0.996	0.996
		RMSE	0.107	0.415	0.153	0.520
	Elovich	*α* (mg/g/min)	0.052	3.457	4.785	0.987
		*β* (g/mg)	1.654	0.565	0.574	0.396
		*R*^2^	0.906	0.915	0.949	0.955
		*R*_adj_^2^	0.902	0.911	0.948	0.953
		RMSE	1.283	0.788	1.018	0.976
	Intraparticle diffusion	*k*_i_ (mg/g min^0.5^)	0.153	0.451	0.450	0.875
		*C*_i_ (mg/g)	0.510	2.987	1.815	8.013
		*R*^2^	0.869	0.816	0.910	0.736
		*R*_adj_^2^	0.864	0.809	0.907	0.727
		RMSE	0.603	2.666	1.564	4.391

For the linear pseudo-first-order kinetic model, the adsorption capacities (*q*_e_) of SB, SBF, SBB, and SBFB were 5.204, 13.722, 8.549, and 34.460 mg/g, and the reaction of rate constants (*k*_1_) of SB, SBF, SBB, and SBFB were 0.014, 0.020, 0.010, and 0.025 min^−1^. For the nonlinear pseudo-first-order kinetic model, the adsorption capacities (*q*_e_) of SB, SBF, SBB, and SBFB were 5.616, 14.806, 8.915, and 34.595 mg/g, and the reaction of rate constants (*k*_1_) of SB, SBF, SBB, and SBFB were 0.016, 0.025, 0.013, and 0.024 min^−1^. For the linear pseudo-second-order kinetic model, the adsorption capacities (*q*_e_) of SB, SBF, SBB, and SBFB were 3.934, 11.455, 10.965, and 21.053 mg/g, and the reaction of rate constants (*k*_2_) of SB, SBF, SBB, and SBFB were 0.003, 0.002, 0.002, and 0.003 g/mg min. For the nonlinear pseudo-second-order kinetic model, the adsorption capacities (*q*_e_) of SB, SBF, SBB, and SBFB were 3.946, 11.903, 11.300, and 21.471 mg/g, and the reaction of rate constants (*k*_2_) of SB, SBF, SBB, and SBFB were 0.003, 0.001, 0.001, and 0.002 g/mg min. For the linear elovich model, the initial adsorption rates (α) of SB, SBF, SBB, and SBFB were 0.047, 3.334, 4.354, and 0.918 mg/g/min, and the extents of surface coverage (*β*) of SB, SBF, SBB, and SBFB were 1.510, 0.494, 0.529, and 0.289 g/mg. For the nonlinear elovich model, the initial adsorption rates (*α*) of SB, SBF, SBB, and SBFB were 0.052, 3.457, 4.785, and 0.987 mg/g/min, and the extents of surface coverage (*β*) of SB, SBF, SBB, and SBFB were 1.654, 0.565, 0.574, and 0.396 g/mg. For the linear intraparticle diffusion model, the reaction of rate constants (*k*_i_) of SB, SBF, SBB, and SBFB were 0.146, 0.444, 0.430, and 0.704 mg/g min^0.5^, and the constant *C*_i_ values of SB, SBF, SBB, and SBFB were 0.490, 2.276, 1.756, and 7.974 mg/g. For the nonlinear intraparticle diffusion model, the reaction of rate constants (*k*_i_) of SB, SBF, SBB, and SBFB were 0.153, 0.451, 0.450, and 0.875 mg/g min^0.5^, and the constant *C*_i_ values of SB, SBF, SBB, and SBFB were 0.510, 2.987, 1.815 and 8.013 mg/g.

For *R*^2^ value consideration, the *R*^2^ values of SB, SBF, SBB, and SBFB in the linear pseudo-first-order kinetic model were 0.911, 0.979, 0.975, and 0.968, and their *R*^2^ values in the linear pseudo-second-order kinetic model were 0.991, 0.992, 0.992, and 0.998. In addition, the *R*^2^ values of SB, SBF, SBB, and SBFB in the linear elovich model were 0.901, 0.912, 0.946, and 0.954, and their *R*^2^ values in the linear intraparticle diffusion model were 0.867, 0.814, 0.906, and 0.730. In addition, the *R*^2^ values of SB, SBF, SBB, and SBFB in the nonlinear pseudo-first-order kinetic model were 0.913, 0.977, 0.977, and 0.971, and their *R*^2^ values in the nonlinear pseudo-second-order kinetic model were 0.993, 0.995, 0.996, and 0.996. Moreover, the *R*^2^ values of SB, SBF, SBB, and SBFB in the nonlinear elovich model were 0.906, 0.915, 0.949, and 0.955, and their *R*^2^ values in the nonlinear intraparticle diffusion model were 0.869, 0.816, 0.910, and 0.736. Moreover, the *R*_adj_^2^ values of SB, SBF, SBB, and SBFB in the nonlinear pseudo-first-order kinetic model were 0.910, 0.976, 0.976, and 0.970, and their *R*_adj_^2^ values in the nonlinear pseudo-second-order kinetic model were 0.992, 0.994, 0.996, and 0.996. In addition, the *R*_adj_^2^ values of SB, SBF, SBB, and SBFB in the nonlinear elovich model were 0.902, 0.911, 0.948, and 0.953, and their *R*_adj_^2^ values in the nonlinear intraparticle diffusion model were 0.864, 0.809, 0.907, and 0.727. Since the *R*^2^ values of SB, SBF, SBB, and SBFB in both linear and nonlinear pseudo-second-order kinetic models were higher than pseudo-first-order kinetic, elovich, and intraparticle diffusion models, so their adsorption rate and mechanism of all sugarcane bagasse adsorbent materials corresponded to pseudo-second-order kinetic model with relating to a chemisorption process with heterogenous adsorption with corresponding to the results of previous studies used sugarcane bagasse for removing lead, nickel, reactive blue 4 dye, and congo red^9,39,40,56^. Therefore, the adsorption capacity (*q*_e_) and the pseudo-second-order kinetic rate constant (*k*_2_) were used for describing the rate and mechanism of all sugarcane adsorbent materials. In Table 5, SBFB had the highest *q*_e_ value than other materials, and it could be arranged from high to low of SBFB > SBF > SBB > SB. For *k*_2_, they had a close value to each other in a range of 0.001–0.003 g/mg min, so it had no significantly different in the kinetic rate constant for lead adsorption on all materials. Finally, the graph plotting of both linear and nonlinear kinetic models was also recommended for correct data translations^40,41,49,50^.

### Desorption experiments

**Figure 9:**
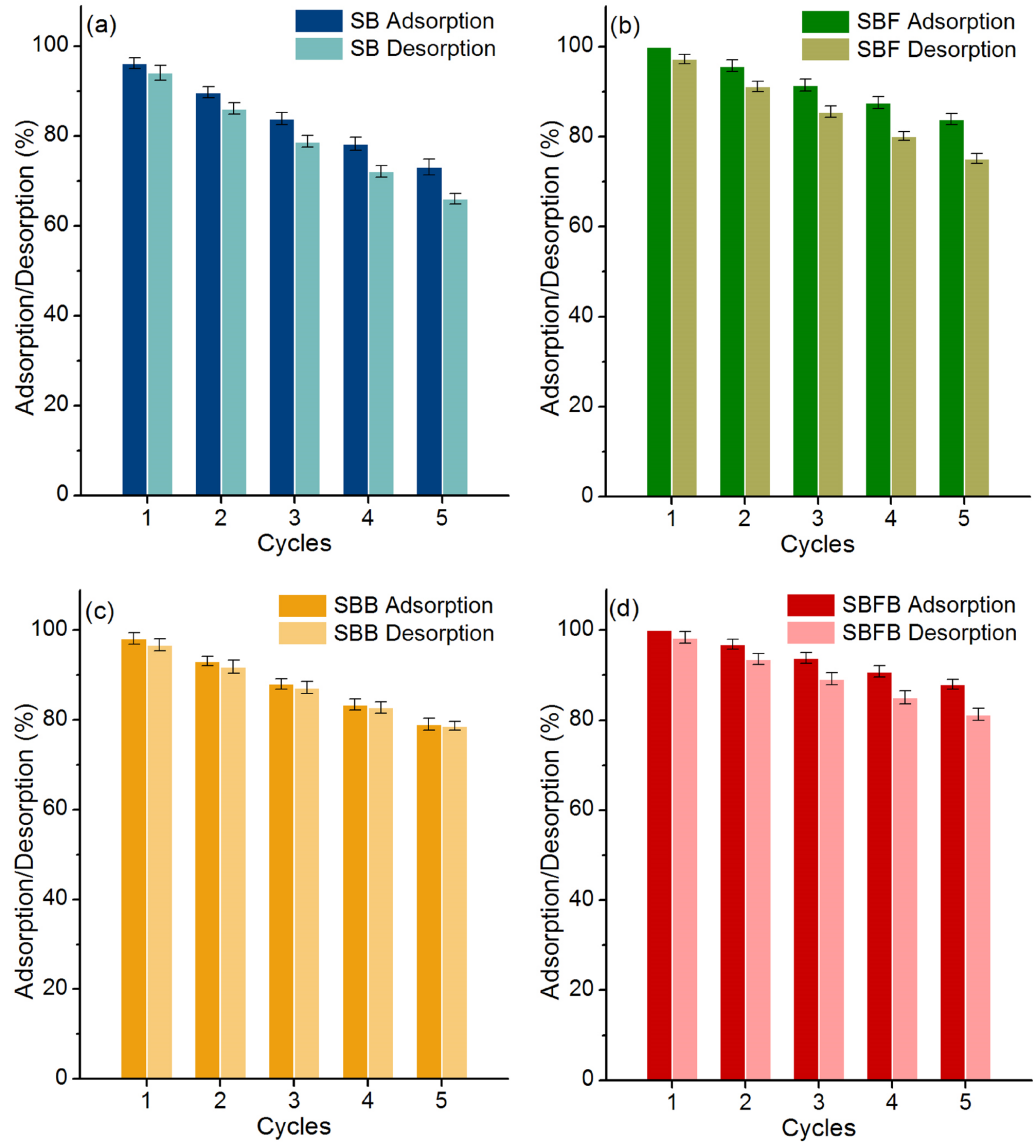
The desorption experiments of (**a**) sugarcane bagasse powder (SB), (**b**) sugarcane bagasse powder doped iron(III) oxide-hydroxide (SBF), (**c**) sugarcane bagasse powder beads (SBB), and (**d**) sugarcane bagasse powder doped iron(III) oxide-hydroxide beads (SBFB) for lead adsorptions.

The desorption experiments are designed to examine the reusability of sugarcane bagasse adsorbent materials for lead adsorptions to predict the investment budget and the feasibility of industrial applications from the economic view. Five adsorption–desorption cycles were applied for investigating the possible reusability of sugarcane bagasse adsorbent materials and their results are demonstrated in Fig. 9a–d. For SB, it could be reused in 5 cycles in ranges of adsorption at 73.11–96.15% and desorption at 66.02–94.07% with the decreasing of adsorption and desorption by 23% and 28%, respectively shown in Fig. 9a. In Fig. 9b, SBF could be reused in 5 cycles in ranges of adsorption at 83.92–100% and desorption at 75.12–97.24% with the decreasing of adsorption and desorption by 16% and 22%, respectively. For SBB, it could be reused in 5 cycles in ranges of adsorption at 79.08–98.19% and desorption at 71.34–95.47% with the decreasing of adsorption and desorption by 19% and 24%, respectively illustrated in Fig. 9c. In Fig. 9d, SBFB could be reused in 5 cycles in ranges of adsorption at 87.96–100% and desorption at 81.27–98.37% with the decreasing of adsorption and desorption by 12% and 17%, respectively. Therefore, all sugarcane adsorption materials could be reused in more than 5 cycles for lead adsorptions, and they are potential materials for further industrial applications.

### The application and environmental feasibility of materials

This study presented four new biosorbent materials from sugarcane bagasse including two material forms of powder and bead forms as choices of users for lead adsorption in wastewater. Four synthesized sugarcane adsorbent materials of SB, SBF, SBB, and SBFB demonstrated high lead adsorptions of more than 96%, so all materials are potential adsorbents for lead removal in an aqueous solution. However, the operation cost might be a significant point for user consideration in industrial applications. Since SBB and SBFB are bead materials, they are easier separate from treated wastewater than SB and SBF. Especially, SBFB is a good option than SBB because it spent less material dosage and contact time than SBB for 100% lead removal including it could be reused more than 5 cycles with a low adsorption capacity loss of approximately 13%. Moreover, sugarcane bagasse is a large amount of waste from sugar factories in Thailand. Therefore, if the sugarcane bagasse can be utilized by synthesizing biosorbent for lead removal, it may be another way to utilize waste for reducing waste management and help reduce lead contamination in the wastewater in another way as well. Therefore, SBFB might be a potential material for applying industrial applications for lead adsorption in the future.

## The possible mechanism of lead adsorption by sugarcane bagasse adsorbent materials

**Figure 10:**
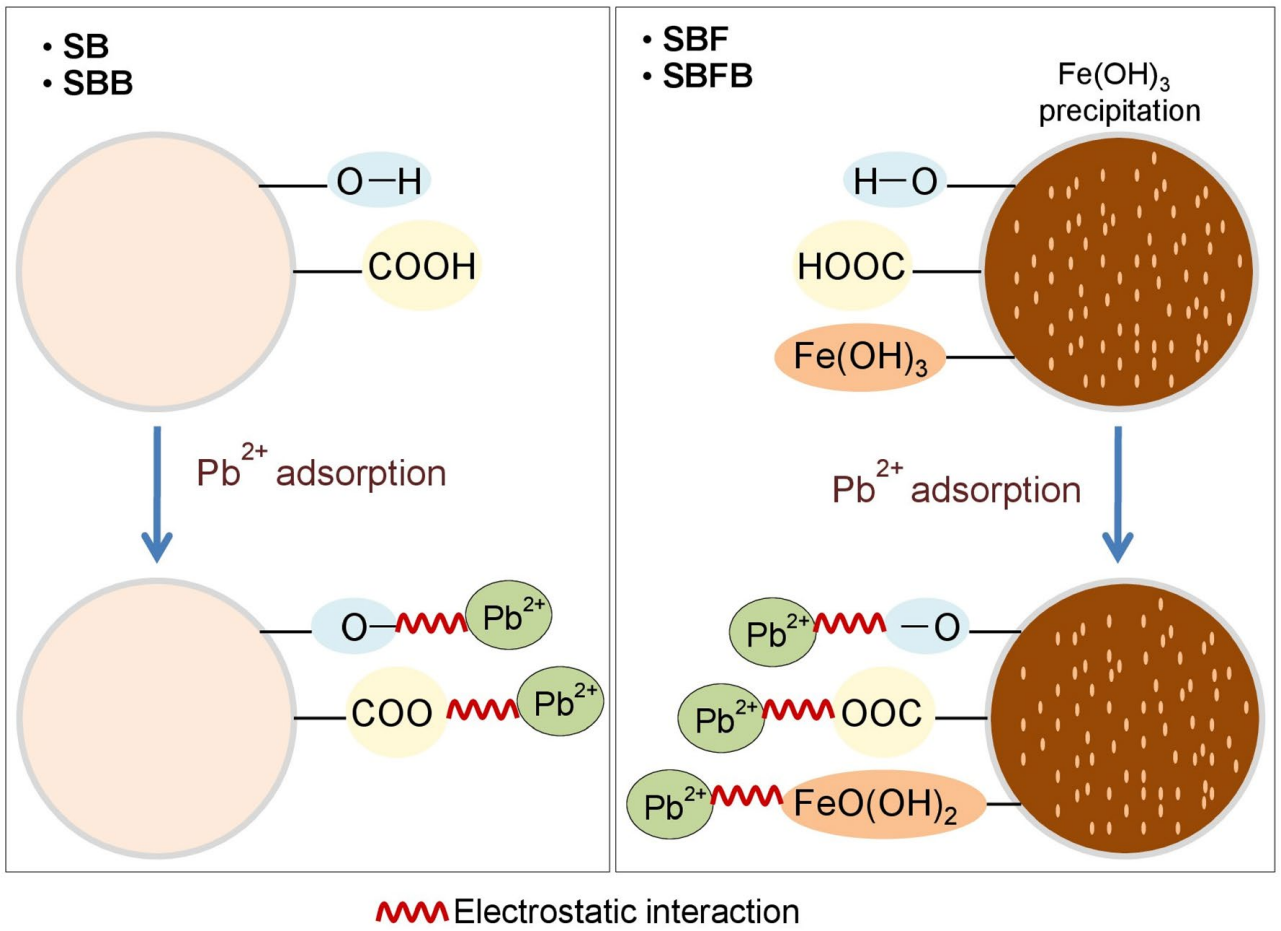
Possible mechanism of lead adsorption on sugarcane bagasse powder (SB), sugarcane bagasse powder doped iron(III) oxide-hydroxide (SBF), sugarcane bagasse powder beads (SBB), and sugarcane bagasse powder doped iron(III) oxide-hydroxide beads (SBFB).

The possible mechanism of lead adsorption on SB, SBF, SBB, and SBFB was explained in Fig. 10. The main structure of sugarcane bagasse adsorbent materials is composed of cellulose, hemicellulose, and lignin confirmed by detecting the specific cellulose peaks represented in XRD analysis. Moreover, the main chemical functional groups of contained carboxyl groups (–COOH) or hydroxyl groups (–OH) were found in all materials by FT-IR analysis. When SB was added by iron(III) oxide-hydroxide to be SBF, they formed the complex compound of iron(III) oxide-hydroxide precipitation on the surface of SB in form of SB·Fe(OH)_3_ by sharing electrons with hydroxyl groups of sugarcane bagasse. In addition, SBB formed the complex compound of SB and sodium alginate whose main structure had a carboxyl group (–COOH) similar to the main compounds of sugarcane bagasse materials. For SBFB, it formed a complex compound by adding iron(III) oxide-hydroxide and sodium alginate similar to SBF and SBB. The possible mechanism of lead adsorptions by sugarcane bagasse adsorbent materials might occur from donating a proton (H^+^) from carboxyl groups (–COOH) or hydroxyl groups (–OH) or SB∙Fe(OH)_3_ of main chemical compounds or complex compounds to be –COO or –O or FeO(OH)_2_ for capturing lead(II) ions (Pb^2+^) by instead of H^+^ from a process of electrostatic interaction^53^. Especially, the pH of the solution (pH_solution_) might affect how much lead is absorbed by the adsorbent. The previous studies found high lead adsorption in pH_solution_ > pH_pzc_ and reported in a range of pH 4–6^9,33,45^ which corresponded to high lead removal efficiencies of all sugarcane adsorbent materials at pH 5 in this study from the batch experiments. Moreover, these results are supported by the results of pH_pzc_ of all materials at pH > 4.

## Conclusion

This study successfully synthesized sugarcane bagasse powder (SB), sugarcane bagasse powder doped iron(III) oxide-hydroxide (SBF), sugarcane bagasse powder beads (SBB), and sugarcane bagasse powder doped iron(III) oxide-hydroxide beads (SBFB). The crystalline patterns of all sugarcane bagasse adsorbent materials were amorphous phases presenting specific peaks of cellulose. The sodium alginate peaks were detected in SBB and SBFB whereas iron(III) oxide-hydroxide peaks were only found in SBF and SBFB. The surface morphologies of SB and SBF were scales or overlapping plate surfaces whereas SBB and SBFB had spherical shapes with coarse surfaces. The main chemical compositions of all sugarcane bagasse adsorbent materials were C, O, and Ca, and Na and Cl were detected in all materials except SB. Fe was only found in SBF and SBFB because of adding iron(III) oxide-hydroxide to raw materials. Five main chemical functional groups of all materials were O–H, C=O, C–H, C–O, and C=C, whereas Fe–O and –COOH were only found in materials with adding iron(III) oxide-hydroxide or bead material. The pH_pzc_ of SB, SBF, SBB, and SBFB were 4.33, 4.88, 4.12, and 4.56, respectively. For batch experiments, the optimum conditions of SB, SBF, SBB, and SBFB were 0.6 g, 6 h, pH 5, 10 mg/L, 0.2 g, 3 h, pH5, 10 mg/L, 0.2 g, 2 h, pH 5, 10 mg/L, and 0.1 g, 2 h, pH 5, 10 mg/L, respectively. Lead removal efficiencies of SB, SBF, SBB, and SBFB of 10 mg/L were 96.08%, 100%, 98.22%, and 100%, respectively, and it could be arranged in order from high to low of SBFB > SBF > SBB > SB. Thus, adding iron(III) oxide-hydroxide and changing material form helped to improve material efficiencies for lead adsorptions. For adsorption isotherms, SB and SBB corresponded to the Langmuir model related to a physical adsorption process whereas SBF and SBFB corresponded to the Freundlich model correlated to a physicochemical adsorption process. For the kinetic study, they corresponded to a pseudo-second-order kinetic model related to a chemisorption process with heterogeneous adsorption. All materials could be reused for more than 5 cycles with a lead removal efficiency of more than 73%. Therefore, all sugarcane bagasse adsorbent materials were high potential materials for lead adsorptions in an aqueous solution, and SBFB demonstrated the highest lead removal efficiency. Therefore, SBFB was a suitable material to further apply to the wastewater treatment system of industry.

For future works, the real wastewater with contaminated lead should be investigated to confirm the ability of sugarcane bagasse materials, and the continuous flow study also needs to study for further industrial applications.

## Materials and methods

### Raw material

Sugarcane bagasse was obtained from a local market in Khon Kaen province, Thailand.

### Chemicals

All chemicals were analytical grades (AR) without purification before use. For modified bead materials, ferric chloride hexahydrate (FeCl_3_·6H_2_O) (LOBA, India), sodium hydroxide (NaOH) (RCI Labscan, Thailand), sodium alginate (NaC_6_H_7_O_6_) (Merck, Germany), and calcium chloride (CaCl_2_) (Kemaus, New Zealand) were used. Lead nitrate (Pb(NO_3_)_2_) (QRëC, New Zealand) was used for preparing of wastewater sample. For the point of zero charge (pH_pzc_) experiment, 0.1 M hydrochloric acid (HCl) (RCI Labscan, Thailand), 0.1 M NaOH and 0.1 M sodium chloride (NaCl) (RCI Labscan, Thailand) solutions were used. For the desorption experiments, a 0.5 M nitric acid (HNO_3_) (Merck, Germany) solution was used. For pH adjustments, 1% HNO_3_ and 1% NaOH solutions were used.

### Synthesis of four adsorbent materials

**Figure 11:**
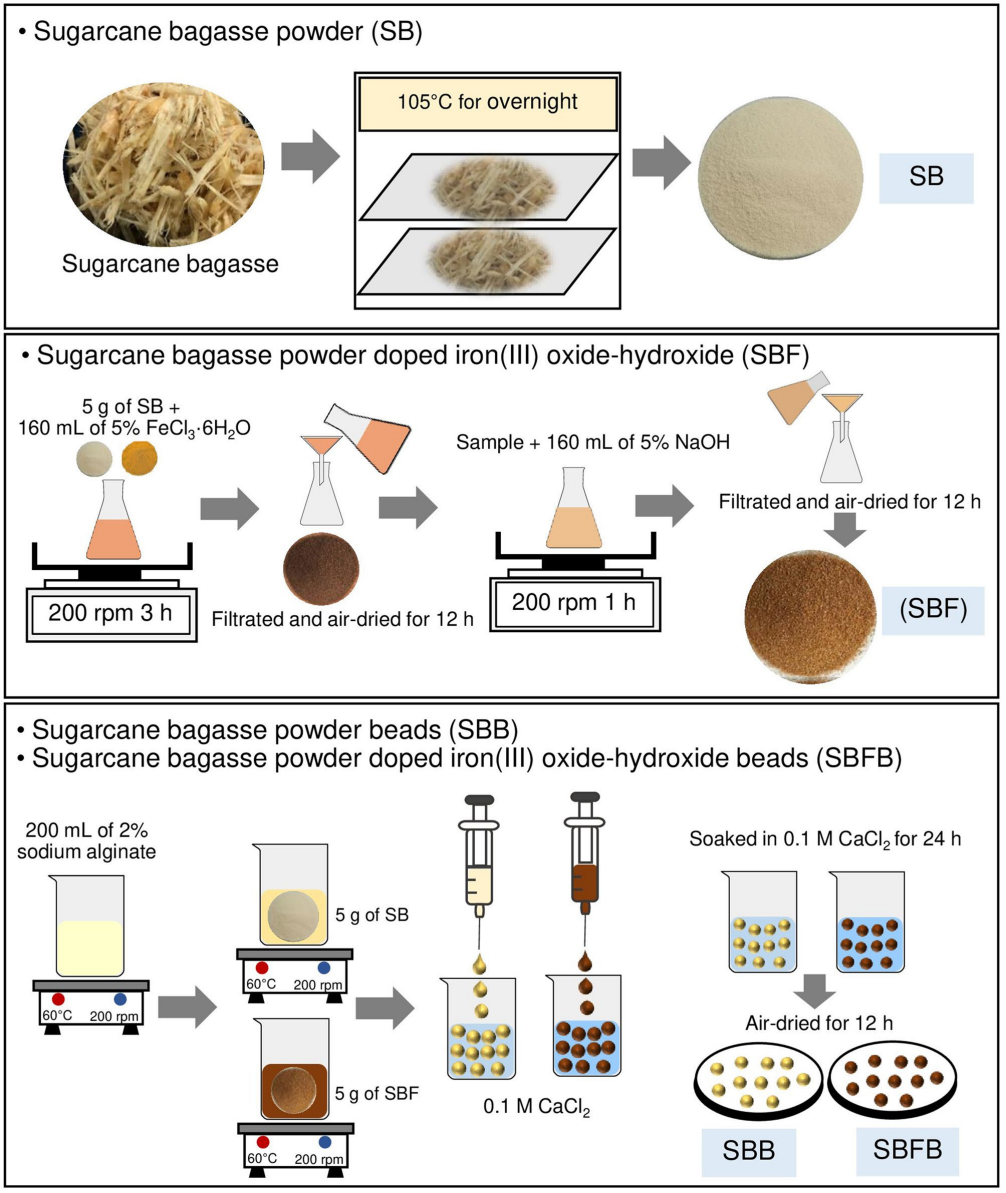
Flow diagrams of synthesis methods of sugarcane bagasse powder (SB), sugarcane bagasse powder doped iron(III) oxide-hydroxide (SBF), sugarcane bagasse powder beads (SBB), and sugarcane bagasse powder doped iron(III) oxide-hydroxide beads (SBFB).

The synthesis of four adsorbent materials which were sugarcane bagasse powder (SB), sugarcane bagasse powder doped iron(III) oxide-hydroxide (SBF), sugarcane bagasse powder beads (SBB), and sugarcane bagasse powder doped iron(III) oxide-hydroxide beads (SBFB) were demonstrated in Fig. 11, and the details were clearly explained below:

#### The synthesis of SB

Sugarcane bagasse was washed with tap water to remove contaminants, and then it was dried overnight in a hot air oven (Binder, FED 53, Germany) at 105 °C. It was ground and sieved in size of 125 µm and kept in a desiccator before use called sugarcane bagasse powder (SB).

#### The synthesis of SBF

SB of 5 g was added to 500 mL of Erlenmeyer flask containing 160 mL of 5% FeCl_3_·6H_2_O, and it was mixed by an orbital shaker (GFL, 3020, Germany) of 200 rpm for 3 h. Then, it was filtrated and air-dried at room temperature for 12 h. After that, it was added to 500 mL of Erlenmeyer flask containing 160 mL of 5% NaOH, and it was mixed by an orbital shaker of 200 rpm for 1 h. Then, it was filtered, air-dried at room temperature for 12 h, and kept in a desiccator before use called sugarcane bagasse powder doped iron(III) oxide-hydroxide (SBF).

#### The synthesis of SBB or SBFB

SB or SBF of 5 g was added to 500 mL of beaker containing 200 mL of 2% sodium alginate, and then it was homogeneously mixed and heated by a hot plate (Ingenieurbüro CAT, M. Zipperer GmbH, M 6, Germany) at 60 °C with a constant stirring of 200 rpm. Then, it was dropped by drop by using a 10 mL syringe with a needle size of 1.2 × 40 mm into 250 mL of 0.1 M CaCl_2_. The beaded samples were soaked in 0.1 M CaCl_2_ for 24 h, and then these were filtered and rinsed with DI water. After that, these were air-dried at room temperature for 12 h and kept in a desiccator before use called sugarcane bagasse powder beads (SBB) or sugarcane bagasse doped iron(III) oxide-hydroxide beads (SBFB).

### Characterizations of sugarcane bagasse adsorbent materials

The crystalline structures, surface morphologies, chemical compositions, and chemical functional groups of SB, SBF, SBB, and SBFB were investigated by X-Ray Diffractometer (XRD) (PANalytical, EMPYREAN, United Kingdom)in the range of 2θ = 5°–60°, Field Emission Scanning Electron Microscopy and Focus Ion Beam (FESEM-FIB) with Energy Dispersive X-Ray Spectrometer (EDX) (FEI, Helios NanoLab G3 CX, USA, and Fourier Transform Infrared Spectroscopy (FT-IR) (Bruker, TENSOR27, Hong Kong), respectively.

### The point of zero charges of sugarcane bagasse adsorbent materials

The experiment of the point of zero charges of SB, SBF, SBB, and SBFB for lead adsorptions is applied from the study of Praipipat et al.^41^. The sample solutions of 0.1 M NaCl at pH values from 2 to 12 were prepared, and 0.1 M HCl and 0.1 M NaOH solutions were used for pH adjustments. Next, the material of 0.1 g was added to a 250 mL Erlenmeyer flask containing each sample solution of 50 mL, and it was shaken by an orbital shaker (GFL, 3020, Germany) at room temperature at 150 rpm for 24 h. After that, a final pH value was measured by (Mettler Toledo, SevenGo with InLab 413/IP67, Switzerland). Then, the ∆pH (pH_final_–pH_initial_) was calculated, and the point of zero charge (pH_pzc_) was determined from a value point of the crosse line of ∆pH versus pH_initial_ equal to zero.

### Batch adsorption experiments

A series of batch adsorption experiments were designed to investigate the effect of dose, contact time, pH, and concentration on lead removal efficiency by SB, SBF, SBB, and SBFB. The difference of dose from 0.1 to 0.6 g, contact time from 1 to 6 h, pH values of 1, 3, 5, 7, 9, 11, and lead concentration from 5 to 30 mg/L with the control condition of initial lead concentration of 10 mg/L, a sample volume of 200 mL, a shaking speed of 200 rpm, a temperature of 25 °C were applied. The lowest value of each affecting factor with the highest lead removal efficiency was selected as the optimum value, and that value was applied to the next affecting factor study. Lead concentrations were analyzed by an atomic adsorption spectrophotometer (PerkinElmer, PinAAcle 900 F, USA), and triplicate experiments were conducted to confirm the results. Lead removal in the percentage (%) is calculated by following Eq. (1).

1 $${\text{Lead\,removal\,efficiency}}\,{\text{(\% )}} = (C_{0} - C_{{\text{e}}} )/C_{0} \times {1}00$$

where *C*_0_ is the initial lead concentration (mg/L), and *C*_e_ is the equilibrium of lead concentration in the solution (mg/L).

### Adsorption isotherms

Adsorption isotherms are designed to identify the adsorption pattern of adsorbent material which may be explained by the adsorption process of monolayer or multi-layer or heat or thermodynamic. Linear and nonlinear Langmuir, Freundlich, Temkin, and Dubinin–Radushkevich models are used to analyze followed Eqs. (2)–(9)^57–60^.

Langmuir isotherm:

2 $${\text{Linear}}{:}\,C_{{\text{e}}} /q_{{\text{e}}} = 1/q_{{\text{m}}} K_{{\text{L}}} + C_{{\text{e}}} /q_{{\text{m}}}$$

3 $${\text{Nonlinear}}{:}\,q_{e} = q_{{\text{m}}} K_{{\text{L}}} C_{{\text{e}}} /{1} + K_{{\text{L}}} C_{{\text{e}}}$$

Freundlich isotherm:

4 $${\text{Linear}}{:}\,{\text{log}}q_{{\text{e}}} = {\text{log}}K_{{\text{F}}} + {1}/n{\text{log}}C_{{\text{e}}}$$

5 $${\text{Nonlinear}}{:}\,q_{{\text{e}}} = K_{{\text{F}}} C_{{\text{e}}}^{{{1}/n}}$$

Temkin isotherm:

6 $${\text{Linear}}{:}\,q_{e} = RT/b_{{\text{T}}} {\text{ln}}A_{{\text{T}}} + RT/b_{{\text{T}}} {\text{ln}}C{\text{e}}$$

7 $${\text{Nonlinear}}{:}\,q_{{\text{e}}} = RT/b_{{\text{T}}} {\text{ln}}A_{{\text{T}}} C_{{\text{e}}}$$

Dubinin–Radushkevich isotherm:

8 $${\text{Linear}}{:}\,{\text{ln}}q_{{\text{e}}} = {\text{ln}}q_{{\text{m}}} {-}K_{{{\text{DR}}}} \varepsilon^{{2}}$$

9 $${\text{Nonlinear}}:q_{e} = q_{{\text{m}}} {\text{exp}}( - K_{{{\text{DR}}}} \varepsilon^{{2}} )$$

where *C*_e_ is the equilibrium of lead concentration (mg/L), *q*_e_ is the amount of adsorbed lead on adsorbent materials (mg/g), *q*_m_ is indicated the maximum amount of lead adsorption on adsorbent materials (mg/g), *K*_L_ is the adsorption constant (L/mg). *K*_F_ is the constant of adsorption capacity (mg/g) (L/mg)^1/n^, and* 1/n* is the constant depicting the adsorption intensity^61^. *R* is the universal gas constant (8.314 J/mol K), *T* is the absolute temperature (K), *b*_T_ is the constant related to the heat of adsorption (J/mol), and *A*_T_ is the equilibrium binding constant corresponding to the maximum binding energy (L/g). *q*_m_ is the theoretical saturation adsorption capacity (mg/g), *K*_DR_ is the activity coefficient related to mean adsorption energy (mol^2^/J^2^), and *ε* is the Polanyi potential (J/mol). Graphs of linear Langmuir, Freundlich, Temkin, and Dubinin–Radushkevich isotherms were plotted by *C*_e_*/q*_e_ versus *C*_e,_ log *q*_e_ versus log* C*_e_, *q*_e_ versus ln* C*_e_, and ln *q*_e_ versus *ε*^2^, respectively whereas graphs of their nonlinear were plotted by *q*_e_ versus *C*_e_*.*

For adsorption isotherm experiments, 0.6 g of SB or 0.2 g of SBF or 0.2 g of SBB, or 0.1 g of SBFB were added to 500 mL Erlenmeyer flask with variable lead concentrations from 5 to 30 mg/L. The control condition of SB, SBF, SBB, and SBFB was a sample volume of 200 mL, a shaking speed of 200 rpm, pH 5, a temperature of 25 °C, and a contact time of 6 h for SB, 3 h for SBB, and 2 h for SBF and SBFB.

### Adsorption kinetics

Adsorption kinetics are studied to investigate the adsorption mechanism of adsorbent material, and linear and nonlinear pseudo-first-order kinetic, pseudo-second-order kinetic, elovich, and intraparticle diffusion models were used following Eqs. (10)–(16) ^62–65^.

Pseudo-first-order kinetic model:

10 $${\text{Linear}}:{\text{ln}}\left( {q_{{\text{e}}} - q_{{\text{t}}} } \right) = {\text{ln}}q_{{\text{e}}} {-}k_{{1}} t$$

11 $${\text{Nonlinear}}{:}\,q_{{\text{t}}} = q_{{\text{e}}} ({1} - e^{{ - k_{1} t}} )$$

Pseudo-second-order kinetic model:

12 $${\text{Linear}}:t/q_{{\text{t}}} = {1}/k_{{2}} q_{{\text{e}}}^{{2}} + \left( {t/q_{{\text{e}}} } \right)$$

13 $${\text{Nonlinear}}{:}\,q_{{\text{t}}} = k_{{2}} q_{{\text{e}}}^{{2}} t/\left( {{1} + q_{{\text{e}}} k_{{2}} t} \right)$$

Elovich model:

14 $${\text{Linear}}:q_{t} = {1}/\beta {\text{ln}}\alpha \beta + {1}/\beta {\text{ln}}t$$

15 $${\text{Nonlinear}}:q_{t} = \beta {\text{ln}}t + \beta {\text{ln}}\alpha$$

Intraparticle diffusion model:

16 $${\text{Linear\,and\,nonlinear}}{:}\,q_{{\text{t}}} = k_{{\text{i}}} t^{{0.{5}}} + C_{{\text{i}}}$$

where *q*_e_ is the amount of adsorbed lead on adsorbent materials (mg/g), *q*_t_ is the amount of adsorbed lead at the time (*t*) (mg/g), *k*_1_ is a pseudo-first-order rate constant (min^−1^), and *k*_2_ is a pseudo-second-order rate constant (g/mg min)^61^. *α* is the initial adsorption rate (mg/g/min) and *β* is the extent of surface coverage (g/mg). *k*_i_ is the intraparticle diffusion rate constant (mg/g min^0.5^) and *C*_i_ is the constant that gives an idea about the thickness of the boundary layer (mg/g). Graphs of linear pseudo-first-order, pseudo-second-order, elovich, and intraparticle diffusion models were plotted by ln (*q*_e_ − *q*_t_) versus time (*t*), *t*/*q*_t_ versus time (*t*), *q*_t_ versus ln *t*, and *q*_t_ versus time (*t*^0.5^), respectively whereas their nonlinear graphs were plotted by the capacity of lead adsorbed by adsorbent materials at the time (*q*_t_) versus time (*t*).

For adsorption kinetic experiments, 3 g of SB or 1 g of SBF or 1 g of SBB, or 0.5 g of SBFB were added to 1000 mL of breaker with a lead concentration of 10 mg/L. The control condition of SB, SBF, SBB, and SBFB was a sample volume of 1000 mL, a shaking speed of 200 rpm, pH 5, a temperature of 25 °C, and a contact time of 8 h.

### Desorption experiments

The possible material reusability is a significant point for considering further industrial applications, so five adsorption–desorption cycles are used for investigating the abilities of SB, SBF, SBB, and SBFB for lead adsorptions through the desorption experiments. The saturated material of SB or SBF or SBB, or SBFB after the adsorption process was added to 500 mL of Erlenmeyer flask containing 200 mL of 0.5 M HNO_3_ solution, and it was shaken by an incubator shaker (New Brunswick, Innova 42, USA) at 200 rpm for 6 h. After that, it was washed with deionization water and dried at room temperature, and then it is ready for the next adsorption cycle. Equation (17) is used for calculating the desorption efficiency in percentage.

17 $${\text{Desorption}}\,{\text{(\% )}} = {(}q_{{\text{d}}} /q_{a} {)} \times {1}00$$

where *q*_*d*_ is the amount of lead desorbed (mg/mL) and *q*_a_ is the amount of lead adsorbed (mg/mL).

## Data Availability

The datasets used and/or analyzed during the current study are available from the corresponding author upon reasonable request.
